# Numerical Investigations on Heat Transfer Characteristics of Mono and Hybrid Nanofluids Using Microchannel Cooling for 21700 Batteries in Electric Vehicles

**DOI:** 10.3390/mi17040497

**Published:** 2026-04-18

**Authors:** Tai Duc Le, Moo-Yeon Lee

**Affiliations:** Department of Mechanical Engineering, Dong-A University, 37 Nakdong-Daero 550, Saha-gu, Busan 49315, Republic of Korea; 2377988@donga.ac.kr

**Keywords:** electric vehicle, hybrid nanofluids, lithium-ion battery, microchannel cooling, thermal management

## Abstract

Efficient thermal management is critical for maintaining the safety, durability, and performance of lithium-ion batteries used in electric vehicles (EVs). In this study, a comprehensive numerical investigation is conducted to evaluate the heat transfer characteristics of mono- and hybrid-nanofluids in a microchannel-cooled lithium-ion battery module. A three-dimensional computational model of a 5S7P battery module composed of cylindrical 21700 cells is developed. Battery heat generation during 3C high discharge rate operation is predicted using the Newman-Tiedemann-Gu-Kim (NTGK) electrochemical model, while coolant flow and heat transfer are simulated using the governing conservation equations for mass, momentum, and energy. The cooling system consists of six liquid-cooling plates with circular microchannels. The performance of water-glycol (50/50) coolant is compared with several mono nanofluids of Al_2_O_3_ and Cu, and hybrid nanofluids of Al_2_O_3_-Cu, Al_2_O_3_-MWCNT, Al_2_O_3_-Graphene, Cu-MWCNT, and Cu-Graphene across multiple coolant flow rates from 1–5 LPM. The results demonstrate that nanofluids significantly enhance convective heat transfer and reduce battery temperature compared with the conventional water-glycol coolant. Among the investigated coolants, the Al_2_O_3_-Cu hybrid nanofluid (0.45–0.45%) operating at 1 LPM achieves the best overall thermo-hydraulic performance with a performance evaluation criterion (PEC) of 1.065. Further analysis of nanoparticle composition ratios shows that a Cu-dominant hybrid mixture (Al_2_O_3_-Cu: 0.27–0.63%) slightly improves the PEC to 1.0657, indicating marginally superior cooling performance. The findings highlight the potential of hybrid nanofluids as advanced coolants for microchannel-based battery thermal management systems in EVs, particularly under moderate coolant flow conditions.

## 1. Introduction

The rapid global transition toward electrified transportation has significantly accelerated the development and deployment of electric vehicles (EVs) as a sustainable alternative to conventional internal combustion engine vehicles [1,2]. Among the various energy storage technologies, lithium-ion batteries (LIBs) have become the dominant choice for EV applications due to their high energy density, long cycle life, low self-discharge rate, and relatively high power density [3,4]. However, during high charge/discharge rates and aggressive driving conditions, LIBs generate considerable heat due to electrochemical reactions, ohmic resistance, and polarization effects [5,6]. If this heat is not effectively dissipated, the battery temperature may rise excessively, leading to accelerated aging, capacity degradation, reduced power capability, and, in severe cases, thermal runaway [7,8]. Consequently, the development of an efficient battery thermal management system (BTMS) is essential to ensure the performance, safety, and reliability of EV battery packs [9,10].

Maintaining an appropriate operating temperature range is critical for LIBs. Previous studies have shown that the optimal operating temperature for LIBs generally lies between 25 °C and 40 °C, while the temperature difference between cells within a module should ideally be maintained below 5 °C to avoid cell imbalance and uneven degradation [11,12,13,14]. Excessive temperature gradients can lead to non-uniform electrochemical reactions, resulting in localized aging and reduced battery lifespan [15,16]. Recent studies have further emphasized that battery temperature strongly affects both electrochemical behavior and system safety. Miao et al. reviewed lithium-ion battery state estimation across a wide temperature range and showed that temperature variations significantly alter internal resistance, capacity, reaction kinetics, and estimation accuracy, underscoring the need for temperature-adaptive battery management [17]. In addition, Kouhestani et al. reported that battery degradation and failure arise from coupled mechanisms and underscored the importance of reliable prognostics and health monitoring for safe EV operation [18]. These findings further motivate the development of effective thermal management strategies that maintain safe, stable battery operation under demanding conditions.

Various BTMS technologies have been proposed in the literature, including air cooling, liquid cooling, phase change material (PCM) cooling, and hybrid cooling strategies [19,20,21]. Among these approaches, liquid cooling systems have demonstrated superior heat removal capability due to liquids’ higher thermal capacity and thermal conductivity compared with air [22,23]. Liquid cooling plates integrated with microchannels have attracted significant attention because they offer a high surface-area-to-volume ratio, enhancing convective heat transfer and enabling compact cooling structures in high-power battery modules [24,25,26]. Microchannel cooling systems are particularly suitable for EV battery packs, where space constraints and high heat generation rates necessitate efficient thermal control.

Despite their advantages, the performance of liquid-cooling systems depends heavily on the coolant’s thermophysical properties. Conventional coolants, such as water or water-glycol mixtures, are widely used in automotive applications because of their chemical stability, low cost, and antifreeze properties [27,28]. However, these fluids often exhibit limited thermal conductivity, which can restrict the overall heat transfer performance of the cooling system [29,30]. To address this limitation, researchers have increasingly explored nanofluids, engineered suspensions of nanoparticles dispersed in a base fluid [31,32]. Nanofluids can exhibit enhanced thermal conductivity and improved heat transfer characteristics compared with conventional fluids [33,34].

Over the past two decades, numerous studies have investigated the thermal performance of mono nanofluids, in which a single type of nanoparticle is dispersed in a base fluid. Nanoparticles such as Al_2_O_3_, Cu, TiO_2_, and SiO_2_ have been widely studied due to their favorable thermal properties and relatively stable dispersion behavior [35,36,37]. More recently, attention has shifted to hybrid nanofluids, which combine two different types of nanoparticles in a single base fluid. The rationale behind hybrid nanofluids is that synergistic interactions among different nanoparticles can potentially yield better thermal performance than mono-nanofluids by simultaneously optimizing thermal conductivity, viscosity, and heat capacity [38,39,40].

Several numerical and experimental studies have examined nanofluids in microchannel heat sinks and electronic cooling applications, demonstrating significant enhancements in heat transfer coefficients and Nusselt numbers [41,42]. In the context of battery thermal management, nanofluids have also been reported to reduce maximum battery temperatures and improve temperature uniformity [43,44,45].

Although significant progress has been made in developing BTMSs using nanofluids, several important research gaps remain. First, many previous studies evaluating nanofluid cooling performance have used pure water as the reference coolant, whereas practical EV thermal management systems typically employ water-glycol mixtures due to their antifreeze capability, corrosion protection, and compatibility with automotive cooling circuits. Consequently, comparisons based solely on water may not accurately reflect real-world cooling conditions in EVs. Second, several existing studies focus primarily on thermal indicators such as maximum temperature reduction or heat transfer coefficient enhancement, but do not comprehensively evaluate the associated hydraulic penalties, which are critical for determining the overall efficiency of a cooling system. Third, while hybrid nanofluids have been shown to potentially outperform mono nanofluids due to synergistic thermophysical effects, the influence of nanoparticle composition ratios within hybrid suspensions remains insufficiently explored, particularly in the context of microchannel-based cooling for cylindrical lithium-ion battery modules. Understanding how different nanoparticle proportions affect thermo-hydraulic behavior could provide valuable guidance for optimizing coolant formulations for microchannel-based BTMS designs. Therefore, the originality of the present work lies not in proposing a fundamentally new BTMS architecture, but in establishing a more application-relevant and comprehensive evaluation of nanofluid cooling for cylindrical lithium-ion battery modules. Specifically, this study benchmarks mono- and hybrid-nanofluids against an automotive water-glycol (50/50) coolant rather than pure water, evaluates both thermal and hydraulic behavior using a unified thermo-hydraulic framework, and further examines the influence of hybrid nanoparticle composition ratio within the same microchannel-cooled 21700 battery module. In this way, the study provides practical design guidance for selecting coolants and optimizing operating conditions in EV-oriented microchannel BTMSs.

To address these research gaps, this study presents a comprehensive numerical investigation of the thermo-hydraulic performance of mono- and hybrid-nanofluids in a microchannel-cooled lithium-ion battery module. A detailed three-dimensional computational model of a 5S7P battery module composed of cylindrical 21700 cells is developed and integrated with a microchannel liquid-cooling plate system to simulate realistic thermal management conditions in EV battery packs. The internal heat generation of the batteries is modeled using the Newman-Tiedemann-Gu-Kim (NTGK) electrochemical model, enabling accurate prediction of heat generation under high discharge rates. Based on this framework, the study systematically compares the thermal and hydraulic performance of a conventional automotive coolant (water-glycol 50/50) with several advanced coolants, including mono nanofluids (Al_2_O_3_ and Cu) and hybrid nanofluids (Al_2_O_3_-Cu, Al_2_O_3_-MWCNT, Al_2_O_3_-Graphene, Cu-MWCNT, and Cu-Graphene). The evaluation is conducted using a comprehensive set of performance indicators that characterize both thermal effectiveness and hydraulic cost, including maximum battery temperature, temperature uniformity within the module, heat transfer coefficient, Nusselt number, pressure drop, friction factor, and the performance evaluation criteria (PEC). Through this systematic comparison, the study identifies the optimal coolant and operating condition for the microchannel cooling system. Furthermore, a parametric analysis is performed to investigate the influence of hybrid nanoparticle composition ratios on overall thermo-hydraulic performance, enabling identification of the most effective hybrid formulation for battery cooling applications. The results provide practical design insights into the potential implementation of nanofluid-based cooling strategies in high-performance BTMSs for EVs, particularly when evaluated against conventional automotive coolants.

The remainder of this paper is organized as follows. Section 2 presents the numerical methodology, including the simulation model, the battery heat generation model, the coolant flow model, and the thermophysical properties of the investigated coolants. Section 3 discusses the simulation results, including comparisons of thermophysical properties, maximum battery temperature, temperature uniformity, heat transfer coefficient, Nusselt number, pressure drop, friction factor, and PEC. In addition, a parametric study on the influence of hybrid nanoparticle composition ratios is conducted to identify the optimal coolant formulation. Finally, Section 4 summarizes the main conclusions and highlights potential directions for future research.

## 2. Methods and Simulation

### 2.1. Simulation Model

The present study investigates the thermal management performance of a liquid-cooled battery module with a 5S7P configuration, consisting of five cells in series and seven cells in parallel. This configuration is representative of compact battery modules commonly used in EVs or energy storage systems, where both thermal uniformity and effective heat dissipation are critical to maintaining performance, reliability, and safety during high-rate operation. Each cell in the module is modeled as a cylindrical 21700 lithium-ion battery with high energy density, favorable thermal characteristics, and robust mechanical structure [46,47]. The principal technical specifications of the 21700 battery used in the numerical model are summarized in Table 1.

The cooling architecture employed in this work consists of six liquid-cooling plates arranged between adjacent rows of cylindrical cells. Each cooling plate integrates one circular microchannel, yielding a total of six microchannels within the battery module. The microchannels have a hydraulic diameter of 3 mm and a channel length of 776 mm, forming a compact network that enhances convective heat transfer while maintaining manageable pressure drops. This microchannel configuration enables the coolant to remove heat effectively from the battery surfaces through conduction across the plate wall and subsequent forced convection within the coolant passages.

The coolant enters the cooling system through inlet ports at the bottom of the cooling plates, flows upward through the microchannels, and exits through outlet ports at the top of the battery module. This upward flow configuration promotes continuous heat removal along the channel length while preventing the accumulation of heated coolant near the inlet region. The total volumetric coolant flow rate, Q, is distributed uniformly across the six parallel cooling plates. Consequently, the flow rate in each microchannel is Q/6, assuming identical channel geometry and uniform flow distribution. This configuration ensures consistent cooling conditions throughout the module and simplifies the analysis of thermo-hydraulic performance.

**Figure 1:**
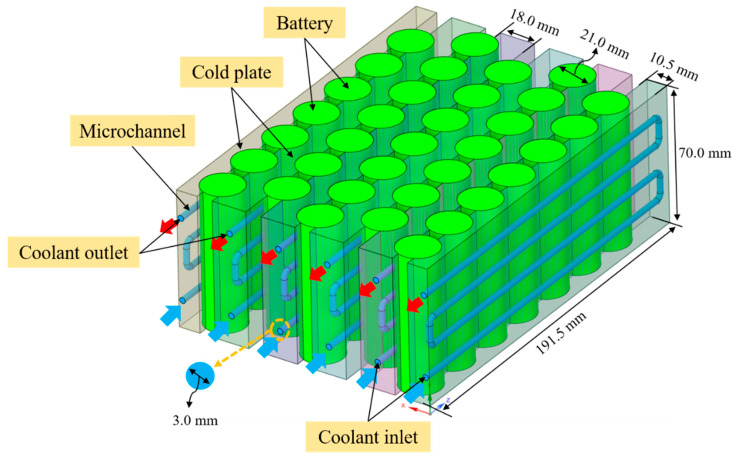
The computational model of the battery module and microchannel cooling structure.

The entire three-dimensional computational model of the battery module and cooling structure is illustrated in Figure 1. The model includes cylindrical battery cells, cooling plates with embedded microchannels, and coolant flow passages. To reduce computational cost while preserving the essential thermal-fluid interactions, geometric features that did not directly influence heat transfer or fluid flow were simplified. The battery cells are treated as solid domains with internal volumetric heat generation determined by the electrochemical heat generation model described in Section 2.2, while the coolant domain is solved using the governing conservation equations for mass, momentum, and energy presented in Section 2.3.

This modeling approach enables detailed evaluation of the coupled thermal-hydraulic behavior of the battery module under high-discharge conditions up to a 3C rate. By combining a realistic module layout with a microchannel-based cooling architecture, the simulation framework provides a representative platform for comparing the thermo-hydraulic performance of various coolants, including conventional water–glycol mixtures and advanced mono- and hybrid-nanofluids.

### 2.2. Battery Heat Generation Model

The thermal behavior of the LIBs during discharge was simulated using a Multi-Scale Multi-Domain (MSMD) framework that incorporated the Newman–Tiedemann–Gu–Kim (NTGK) semi-empirical model. The NTGK model is employed to predict battery heat generation, which has been widely validated for LIBs under various operating conditions, including high discharge rates. At elevated C-rates, nonlinear effects such as concentration polarization and increased ohmic heating become more significant. In the NTGK framework, these effects are incorporated implicitly through experimentally fitted parameters and the dependence of heat generation on current and voltage. While the model does not explicitly resolve detailed electrochemical transport phenomena, it provides a computationally efficient and sufficiently accurate approach for system-level thermal analysis [11,48,49]. It should be noted that although the NTGK model captures the dominant heat generation behavior at high discharge rates, more detailed electrochemical models, such as pseudo-two-dimensional (P2D) models, may provide improved accuracy in resolving internal concentration gradients and polarization effects [50,51]. The NTGK model couples the cell’s macroscopic potential fields with local electrochemical kinetics through a set of fitting parameters, specifically the transfer conductance (Y) and reference voltage (U), which are functions of the depth of discharge (DOD) and local temperature. These parameters were calibrated against experimental voltage-capacity and impedance data to ensure high fidelity across varying C-rates. In the cell-scale energy conservation equation, the total volumetric heat source accounts for Ohmic dissipation in the solid current collectors and electrodes, as well as electrochemical reaction heat, including reversible entropic heat and irreversible polarization losses, thereby capturing the spatial and temporal temperature gradients during discharge [48,49].

To capture the battery’s cell-scale electro-thermal behavior, the computational domain is solved using the coupled electrical and thermal governing equations given below [52]:

(1) $$\frac{\partial (\rho c_{p}T)}{\partial t}-\nabla \cdot (k_{b}\nabla T)={\sigma_{pos}\left|\nabla \Phi_{pos}\right|}^{2}+{\sigma_{neg}\left|\nabla \Phi_{neg}\right|}^{2}+{\dot{q}}_{Ech}+{\dot{q}}_{short}$$

(2) $$\nabla \cdot \left(\sigma_{pos}\nabla \Phi_{pos}\right)=-\left(j_{Ech}-j_{short}\right)$$

(3) $$\nabla \cdot \left(\sigma_{neg}\nabla \Phi_{neg}\right)={(j}_{Ech}-j_{short})$$

where Φ denotes the phase potential, and σ is the effective electrical conductivity of the electrode material. The term ${\dot{q}}_{Ech}$ represents volumetric heat generation associated with electrochemical reactions, while $j_{Ech}$ is the corresponding volumetric current transfer rate. In addition, ${\dot{q}}_{short}$ and $j_{short}$ account for heat release and current transfer arising from internal short-circuit pathways, respectively. The subscripts pos and neg refer to the positive and negative electrodes. In the present work, the source terms $j_{Ech}$ and ${\dot{q}}_{Ech}$ are evaluated using the NTGK electrochemical sub-model, which provides a semi-empirical yet computationally efficient description of cell electrochemistry.

The phase-potential difference is related to the volumetric current transfer rate through the NTGK formulation as [52]:

(4) $$j=\frac{C_{N}}{C_{ref}Vol}Y\left[U-\left(\emptyset_{pos}-\emptyset_{neg}\right)\right]$$

Here, Vol denotes the active-zone volume, and $C_{ref}$ is the reference cell capacity used when fitting the parameter functions U and Y.

The DOD is used as a state variable to track the extracted capacity over the discharge period and is influenced by the volumetric current transfer rate j and the active volume Vol. It can be computed by time-integrating j as [52]:

(5) $$DOD=\frac{Vol}{3600Q_{nominal}}\int_{0}^{t}jdt$$

In the NTGK framework, the fitted functions U and Y are expressed primarily as DOD-dependent polynomials with temperature corrections [53]:

(6) $$U=\left\{\sum_{n=0}^{5}a_{n}{\left(DOD\right)}^{n}\right\}-C_{2}\left(T-T_{ref}\right)\;Y=\left\{\sum_{n=0}^{5}b_{n}{\left(DOD\right)}^{n}\right\}exp\left(-C_{1}\left\{\frac{1}{T}-\frac{1}{T_{ref}}\right\}\right)$$

where $T_{ref}$ is the reference temperature, and $C_{1}$ and $C_{2}$ are the specific constants of the given battery.

The volumetric heat source associated with electrochemical reactions is evaluated as [53]:

(7) $$q_{ech}=j\left[U-\left(\emptyset_{pos}-\emptyset_{neg}\right)-T\frac{dU}{dT}\right]$$

In this expression, the term involving $U-\left(\emptyset_{pos}-\emptyset_{neg}\right)$ corresponds to overpotential heating, whereas the $T\frac{dU}{dT}$ term accounts for the reversible entropic contribution. The NTGK model parameters used in this study were obtained through experimental calibration using the same 21700 LIB (Samsung INR21700-48X, Samsung SDI Co., Ltd., Yongin-si, Republic of Korea). Discharge experiments were conducted at multiple C-rates, including 0.5C (2.4 A), 1.0C (4.8 A), 1.5C (7.2 A), 2.0C (9.6 A), 2.5C (12 A), and 3.0C (14.4 A), under natural convection conditions with an ambient temperature of 25 °C. The measured voltage–time profiles were used as inputs to the NTGK model to determine the fitting parameters U and Y as functions of depth of discharge (DOD). This calibration ensures that the electrochemical heat generation behavior is accurately represented for the specific battery used in this study. The NTGK fitting parameters including the U and Y coefficients are summarized in Table 2. In addition, the thermophysical properties of the battery used in the NTGK model include a density of 2739.62 kg/m^3^, a specific heat capacity of 1605 J/kg·K, and thermal conductivity (x, y, z) of 29/2.6/29 W/m·K.

### 2.3. Coolant Flow Model

In the present work, the microchannel coolant is modeled as water-glycol, mono nanofluids (MNFs), and hybrid nanofluids (HNFs) based on water (base fluid) to quantify the influence of nanoparticle composition on convective heat removal from the 21700 battery module. The MNFs comprise 0.9%Al_2_O_3_-water (MNF1) and 0.9%Cu-water (MNF2), while the HNFs are formulated by combining two nanoparticle types at equal volume fractions, including 0.45%Al_2_O_3_-0.45%Cu-water (HNF1), 0.45%Al_2_O_3_-0.45%MWCNT-water (HNF2), 0.45%Al_2_O_3_-0.45%Graphene-water (HNF3), 0.45%Cu-0.45%MWCNT-water (HNF4), and 0.45%Cu-0.45%Graphene-water (HNF5). In this study, nanofluids are modeled as homogeneous single-phase fluids using effective thermophysical property correlations, enabling consistent comparison of thermohydraulic performance under identical boundary conditions and flow regimes. Although this approach does not explicitly account for particle-fluid interactions, microscale effects such as Brownian motion, particle dispersion, and interfacial thermal transport are implicitly incorporated through the adopted correlations for effective thermal conductivity, viscosity, density, and specific heat. This single-phase framework is widely used for nanofluid simulations at low particle concentrations and provides a reasonable balance between computational efficiency and predictive capability [53,54]. Nevertheless, the single-phase assumption neglects local particle migration and agglomeration effects, which may influence heat transfer and flow behavior in practical applications. Table 3 lists the thermophysical characteristics of water-glycol and nanoparticles (Al_2_O_3_, Cu, Graphene, and MWCNT).

The thermophysical properties of MNFs are calculated as follows:

Density of MNF [54]:

(8) $$\rho_{MNF}=\varphi_{np}\rho_{np}+(1-\varphi_{np})\rho_{bf}$$

Specific heat of MNF [54]:

(9) $${\left(\rho c_{p}\right)}_{MNF}=\varphi_{np}\rho_{np}c_{p,np}+(1-\varphi_{np})({\rho c_{p})}_{bf}$$

Viscosity of MNF [54]:

(10) $$\mu_{MNF}=\mu_{bf}(1+B\varphi_{np}+C{\varphi_{np}}^{2})$$

Thermal conductivity of MNF [54]:

(11) $$\frac{k_{MNF}}{k_{bf}}=\frac{k_{np}+\left(m-1\right)k_{bf}+(m-1)\varphi_{np}(k_{np}-k_{bf})}{k_{np}+\left(m-1\right)k_{bf}-\varphi_{np}(k_{np}-k_{bf})}$$

The thermophysical properties of HNFs are calculated as follows:

Density of HNF [54]:

(12) $$\rho_{HNF}=\varphi_{1}\rho_{1}+\varphi_{2}\rho_{2}+(1-\varphi_{1}-\varphi_{2})\rho_{bf}$$

Specific heat of HNF [54]:

(13) $${\left(\rho c_{p}\right)}_{HNF}=\varphi_{1}{\left(\rho c_{p}\right)}_{1}+\varphi_{2}{\left(\rho c_{p}\right)}_{2}+(1-\varphi_{1}-\varphi_{2})({\rho c_{p})}_{bf}$$

Viscosity of HNF [54]:

(14) $$\varphi \mu_{HNF}=\varphi_{1}\mu_{nf,1}+\varphi_{2}\mu_{nf,2}$$

where:

(15) $$\mu_{nf,i}=\mu_{bf}(1+B_{i}\varphi +C_{i}\varphi^{2})$$

Thermal conductivity of HNF [54]:

(16) $$\varphi k_{HNF}=\varphi_{1}k_{nf,1}+\varphi_{2}k_{nf,2}$$

where:

(17) $$\frac{k_{nf,i}}{k_{bf}}=\frac{k_{p,i}+\left(m_{i}-1\right)k_{bf}+(m_{i}-1)\varphi (k_{p,i}-k_{bf})}{k_{p,i}+\left(m_{i}-1\right)k_{bf}-\varphi (k_{p,i}-k_{bf})}$$

where $\rho_{bf}$, $\mu_{bf}$, $k_{bf}$ represent the density, dynamic viscosity, and thermal conductivity of the base fluid, while $\rho_{np}$, $c_{p,np}$, $k_{np}$ denote the density, specific heat capacity, and thermal conductivity of the nanoparticles. The parameter $\varphi_{np}$ represents the nanoparticle volume fraction dispersed in the base fluid. In the viscosity model, B and C are empirical constants related to nanoparticle shape and interaction effects, while m is a shape factor used in the thermal conductivity correlation. These parameters collectively describe how the addition of nanoparticles modifies the effective thermophysical properties of the mono nanofluid, as reported in the literature [54]. Table 4 presents the calculated thermophysical property values for both mono- and hybrid-nanofluids. In the present study, the thermophysical properties of the base fluid and nanofluids were assumed to be constant and evaluated at a reference temperature of 25 °C. This assumption is reasonable because the temperature variation within the battery module remains relatively small, ranging from 25 to 32 °C. Within this limited range, variations in thermophysical properties are expected to be minor and do not significantly affect the comparative thermo-hydraulic trends among different coolants. Nevertheless, incorporating temperature-dependent thermophysical properties could further improve model fidelity and will be considered in future work.

The coolant flow is governed by the conservation equations of mass, momentum, and energy [55]:

(18) $$\nabla \cdot \vec{u}=0$$

(19) $$\rho_{c}\nabla \cdot \left(\vec{u}\vec{u}\right)+\nabla p-\mu_{c}\nabla \vec{u}=0$$

(20) $$\rho_{c}c_{pc}\frac{{\partial T}_{c}}{\partial t}+\nabla \cdot \left(\rho_{c}c_{pc}\vec{u}T_{c}\right)-\nabla \cdot \left(k_{c}\nabla T_{c}\right)=0$$

where $\rho_{w}$, $\vec{u}$, $\mu_{w}$, p, $c_{pw}$ and $k_{w}$ are the density, velocity vector, dynamic viscosity, static pressure, specific heat capacity and thermal conductivity of coolant, respectively.

### 2.4. Boundary Conditions

Appropriate boundary conditions were applied to accurately simulate the thermo-fluid behavior of the microchannel cooling system and the heat generation within the lithium-ion battery module. In the present study, the coolant enters the microchannel cooling plates through mass flow rate inlet boundaries, ranging from 1 to 5 LPM. The initial temperature of the battery module and coolant is maintained at 25 °C. The outlet of each cooling plate was defined as a pressure outlet boundary condition with atmospheric reference pressure, allowing the coolant to exit the microchannels freely while maintaining numerical stability.

The internal walls of the microchannels were treated as no-slip walls, ensuring that the fluid velocity at the walls was zero. Conjugate heat transfer between the coolant and the battery cells was modeled by coupling the solid and fluid domains, enabling heat generated within the battery cells to be conducted through the cooling plates and subsequently removed by the coolant flow. The battery cells were modeled as solid domains with volumetric heat generation, calculated using the NTGK electrochemical model described in Section 2.2 to account for the electrochemical heat generated during a 3C discharge rate. It should be noted that battery discharge is inherently a transient process. In the present study, a steady-state approach was adopted based on the peak heat generation condition at the 3C discharge rate. This assumption enables evaluation of the cooling system performance under the most critical thermal load. However, the temporal evolution of battery temperature is not captured in this framework. Incorporating fully transient electro-thermal simulations would provide additional insight into time-dependent behavior and will be considered in future work.

The external surfaces of the battery module were assumed to be thermally insulated, implying negligible heat loss to the surrounding environment. This assumption allows the study to focus on the effectiveness of the internal liquid-cooling system without interference from external convective heat losses. The coolant flow in the microchannels was assumed to be laminar, incompressible and single-phase. Based on the hydraulic diameter of 3 mm and the investigated flow rates from 1–5 LPM distributed across six parallel channels, the corresponding Reynolds numbers remain within the laminar regime. Therefore, no turbulence model was employed in the present simulations. In addition, the smooth circular geometry of the microchannels and steady inlet conditions reduce the likelihood of transition to turbulence. Thus, transitional effects are considered negligible. These boundary conditions collectively provide a realistic representation of the operating conditions encountered in liquid-cooled battery thermal management systems for electric vehicles while ensuring numerical stability and computational efficiency.

In addition, the convergence of the numerical solution was assessed using both residual criteria and monitoring of key physical parameters. The residuals for continuity and momentum equations were set to 10^−3^, while a stricter convergence criterion of 10^−6^ was applied to the energy equation to ensure accurate thermal predictions. In addition, key variables, including maximum battery temperature, outlet temperature, and pressure drop, were continuously monitored during iterations. Convergence was assumed when these parameters showed negligible variation, with differences of less than 0.1% across successive iterations.

### 2.5. Data Reduction

The temperature difference within the battery module is quantified as follows [11,52]:

(21) $$∆T=T_{max,battery}-T_{min,battery}$$

where $T_{max,battery}$ represents the highest temperature recorded among the battery cells, while $T_{min,battery}$ corresponds to the lowest cell temperature within the battery module.

The heat transfer coefficient (HTC) is evaluated as follows [11,52]:

(22) $$h=\frac{Q_{conv,coolant}}{A(T_{mean,battery}-T_{mean,coolant})}$$

The mean temperature of the coolant is determined as follows [11,52]:

(23) $$T_{mean,coolant}=\frac{T_{inlet,coolant}+T_{outlet,coolant}}{2}$$

The convective heat transfer from the battery surface to the coolant is estimated as follows [11,52]:

(24) $$Q_{conv,coolant}={\dot{m}}_{coolant}C_{p,coolant}(T_{outlet,coolant}-T_{inlet,coolant})$$

The Nusselt number (Nu) of the coolant is calculated as follows [11,52]:

(25) $${Nu}_{coolant}=\frac{hD_{h}}{k_{coolant}}$$

The pressure drop (Δ*P*) across the cooling channel is determined as follows [11,52]:

(26) $$∆P_{coolant}=P_{inlet,\;coolant}-P_{outlet,coolant}$$

The friction factor of the coolant flow is calculated as follows [11]:

(27) $$f=\frac{2D_{h}\left(P_{inlet,coolant}-P_{outlet,coolant}\right)}{L\rho_{coolant}u^{2}}$$

The performance evaluation criterion (*PEC*) is determined as follows [11]:

(28) $$PEC=\frac{\left(Nu/{Nu}_{0}\right)}{{\left(f/f_{0}\right)}^{\frac{1}{3}}}$$

where A denotes the effective heat transfer area, $T_{mean,battery}$ represents the mean temperature of the battery module, and $T_{mean,coolant}$ denotes the average temperature of the coolant. The symbols $T_{outlet,coolant}$ and $T_{inlet,coolant}$ correspond to the outlet and inlet temperatures of the coolant, respectively. The parameters ${\dot{m}}_{coolant}$, $C_{p,coolant}$, and $\rho_{coolant}$ represent the mass flow rate, specific heat capacity, and density of the coolant, respectively. In addition, $P_{inlet,coolant}$ and $P_{outlet,coolant}$ denote the inlet and outlet pressures of the coolant. The symbol $D_{h}$ represents the hydraulic diameter of the microchannel, L is the length of the cooling channel, u is the coolant velocity, and $k_{coolant}$ denotes the thermal conductivity of the coolant. The subscript 0 refers to the baseline configuration (water/glycol) used for performance comparison.

### 2.6. Mesh Independence Test

The computational mesh for the present study was generated using ANSYS Fluent Meshing 2025 R2 (Ansys Inc., Canonsburg, PA, USA). To accurately capture the thermal and fluid flow characteristics within the battery module and microchannel cooling system, a non-uniform meshing strategy was adopted. Regions near the battery surfaces and at the cooling plate interfaces were refined using local surface sizing, ensuring sufficient spatial resolution to accurately capture heat transfer between the battery cells and the cooling plates. In contrast, the interior regions of the battery solid domain, where temperature gradients are relatively smooth, do not require highly refined elements. Therefore, local body sizing was applied to these regions to reduce the overall mesh count while maintaining adequate numerical accuracy. For surface mesh generation, curvature and proximity size functions were used to automatically refine the mesh in regions with high geometric curvature and narrow gaps between adjacent components, particularly at the cooling plates and battery interfaces. To accurately capture the near-wall velocity and thermal boundary layers, inflation layers were applied along the solid-fluid interfaces in the microchannel domains. This approach improves the resolution of velocity gradients and temperature fields near the walls, which are critical for predicting convective heat transfer performance. The volume mesh was generated using the poly-hexcore meshing method, which combines polyhedral cells near complex geometries with a hexahedral core structure in the bulk fluid region. This hybrid meshing approach provides a good balance between numerical accuracy and computational efficiency by maintaining high-quality cells near intricate surfaces while reducing the number of elements in regions where the flow field is relatively uniform.

**Figure 2:**
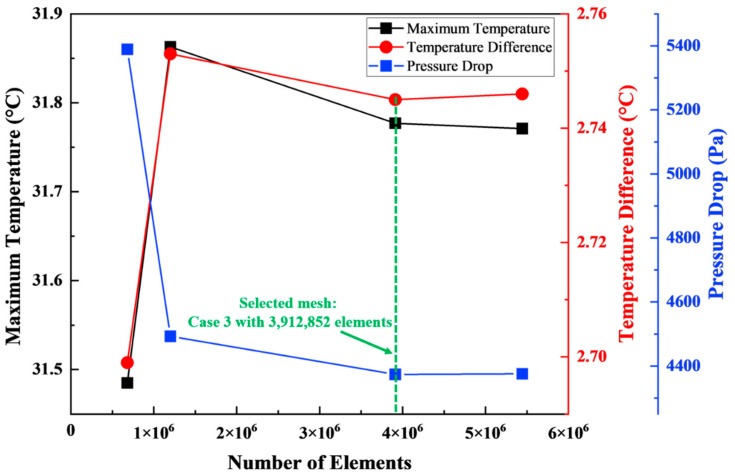
Test of mesh independence.

**Figure 3:**
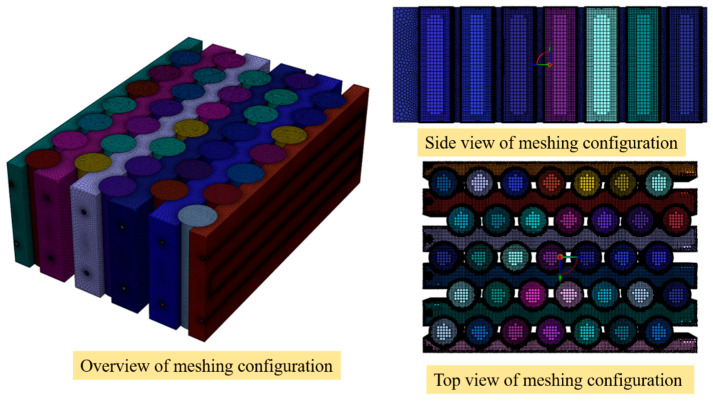
Computational geometry meshing.

To verify the independence of the numerical solution from mesh resolution, a mesh independence study was conducted using four different mesh densities, as illustrated in Figure 2. The tested meshes consisted of 680,929 elements (Case 1), 1,199,243 elements (Case 2), 3,912,852 elements (Case 3), and 5,442,932 elements (Case 4). The effects of mesh density on key performance indicators, including maximum battery temperature, temperature difference within the module, and pressure drop across the cooling channels, were evaluated. As shown in Figure 2, the variation of key parameters with mesh density exhibits clear convergence behavior, further supporting mesh independence. Specifically, the comparison showed that the differences between Case 3 and the finest mesh (Case 4) were extremely small, with deviations of 0.018% for the maximum temperature, 0.036% for the temperature difference, and 0.045% for the pressure drop. These values are well below typical numerical uncertainty thresholds for CFD simulations, indicating that the solution is effectively independent of further mesh refinement. Therefore, Case 3, containing 3,912,852 elements, was selected for the final simulations as it provides a good compromise between solution accuracy and computational cost. Although a formal Grid Convergence Index (GCI) analysis is not included, the monotonic convergence behavior and negligible variation of key parameters across successive mesh refinements confirm that the selected mesh provides sufficient numerical accuracy. The mesh configuration applied in the current numerical simulations is shown in Figure 3.

### 2.7. Experimental and Battery Thermal Model Validation

**Figure 4:**
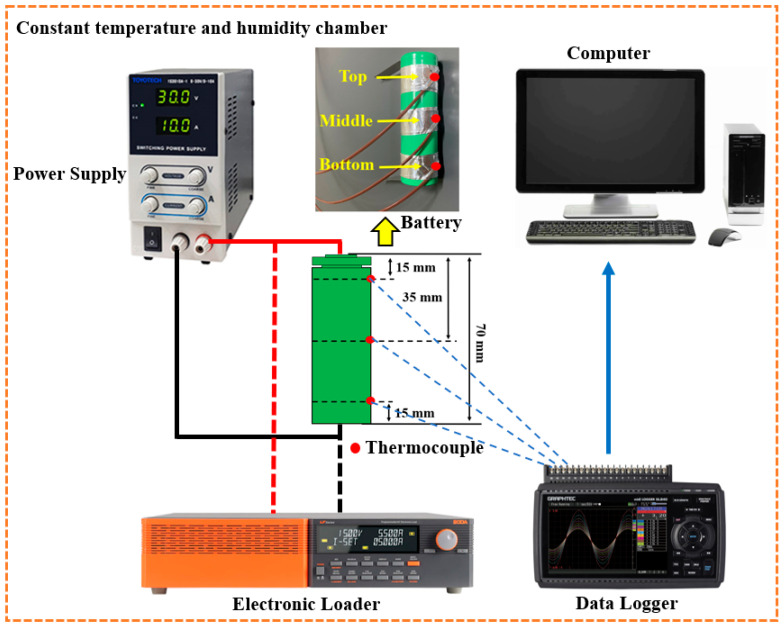
Schematic diagram of battery experiment setup.

Experimental studies were conducted to estimate the parameters of the NTGK model for heat generation prediction and validate the numerical simulation model for evaluating the thermal performance characteristics of the battery. The schematic diagram of the experimental setup is shown in Figure 4.

The battery was discharged using a TLF1200 electronic load (Toyotech Co., Ltd., Incheon, Republic of Korea), with a maximum power of 1200 W, operating in the voltage range of 1 to 150 V and a current range of 0 to 240 A. A TS3010A-1 power supply (Toyotech Co., Ltd., Incheon, Republic of Korea), with a maximum power of 300 W, a voltage range of 0 to 30 V, and a current range of 0 to 10 A, was used to charge the battery. Temperature and voltage data during discharge were recorded using a Graphtec GL840 midi data logger (GRAPHTEC, Yokohama, Japan). All experiments were performed in a controlled environment where the temperature was maintained at 25 °C using a constant temperature and humidity chamber.

In this study, a commercial Samsung INR21700-48X battery was selected for discharge experiments. The battery was subjected to discharge tests at rates of 0.5 C, 1 C, 1.5 C, 2 C, 2.5 C, and 3.0 C with an initial voltage of 4.2 V and a cut-off voltage of 2.5 V. Three T-type thermocouples were attached to the battery surface at different locations, namely the top, middle, and bottom, with an operating temperature range of −220 °C to 400 °C with an accuracy of ±0.1 °C.

For the LIBs to operate at their optimal performance, a uniform temperature distribution across the battery surface is crucial to ensure thermal performance. Therefore, the temperature variation across the cylindrical battery surface should be considered when analyzing its thermal characteristics. During the discharge cycles, the maximum temperature difference between the three monitored locations was always maintained below 1.0 °C. This observation is consistent with the findings of Li et al. [56] and Kong et al. [57], who noted that the temperature difference between the top, middle, and bottom positions of the cylindrical battery is very small. Therefore, the temperature measured at the middle position can be considered as a representative value of the overall temperature of the battery surface. Based on this observation, the numerical simulation results were validated by comparing them with the experimental temperature data obtained from the middle position of the battery.

**Figure 5:**
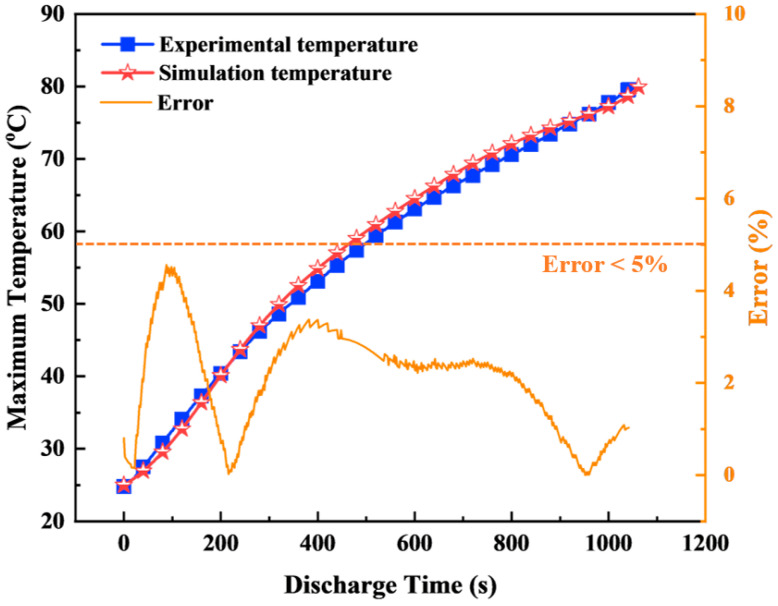
Numerical model validation results with experiments.

Figure 5 shows a comparison between the simulated and experimental results for the temperature of the battery discharge process at a discharge rate of 3C. Simulations of a single cell were performed under natural convection conditions, with the convection coefficient set at 10 W/m^2^·K. The initial temperature of the cell and the ambient temperature were both set at 25 °C. The results show that the percentage error between the simulated and experimental temperatures is always below 5%. The simulation results from the proposed numerical model are consistent with the corresponding experimental data, thus confirming the accuracy of the model. This agreement confirms that the experimentally calibrated NTGK parameters yield reliable heat generation predictions for the 21700 cell at high discharge rates. Therefore, the proposed numerical model is considered suitable for further simulations of the battery module with an indirect cooling system using nanofluid. It should be noted that the current validation is performed at the cell level under natural convection conditions, primarily to verify the accuracy of the electrochemical heat generation model. The coupled thermo-fluid behavior of the full battery module with microchannel liquid cooling and nanofluids is modeled based on established governing equations and validated correlations. However, experimental validation of the complete integrated system is not included in this study and remains a subject for future work.

## 3. Results and Discussion

This section presents a comprehensive analysis of the thermo-hydraulic performance of the investigated coolants for the microchannel-cooled 21700 lithium-ion battery module. Based on the numerical framework described in Section 2, the cooling performance of the conventional water-glycol (50/50) coolant is systematically compared with several mono nanofluids (Al_2_O_3_ and Cu) and hybrid nanofluids (Al_2_O_3_-Cu, Al_2_O_3_-MWCNT, Al_2_O_3_-Graphene, Cu-MWCNT, and Cu-Graphene) over a range of coolant flow rates from 1 to 5 LPM under a 3C discharge condition. The analysis begins with a comparison of the thermophysical properties of the working fluids, which directly influence the cooling system’s heat transfer and hydraulic behavior. Subsequently, the thermal performance of the battery module is evaluated in terms of maximum temperature and temperature difference, followed by an assessment of the convective heat transfer characteristics, including the heat transfer coefficient and Nusselt number. In addition, the hydraulic performance of the cooling system is analyzed using pressure drop and friction factor to quantify the pumping requirements for each coolant. Finally, the performance evaluation criteria (PEC) are employed to provide an integrated thermo-hydraulic assessment that balances heat transfer enhancement against hydraulic penalties. Furthermore, a parametric investigation of hybrid nanoparticle composition ratios is conducted to identify the optimal hybrid formulation for microchannel battery cooling. Through this systematic analysis, the section provides detailed insights into the mechanisms governing nanofluid cooling performance and establishes the most effective coolant configuration for advanced BTMSs in EVs.

### 3.1. Comparison of MNF and HNF Properties

**Figure 6:**
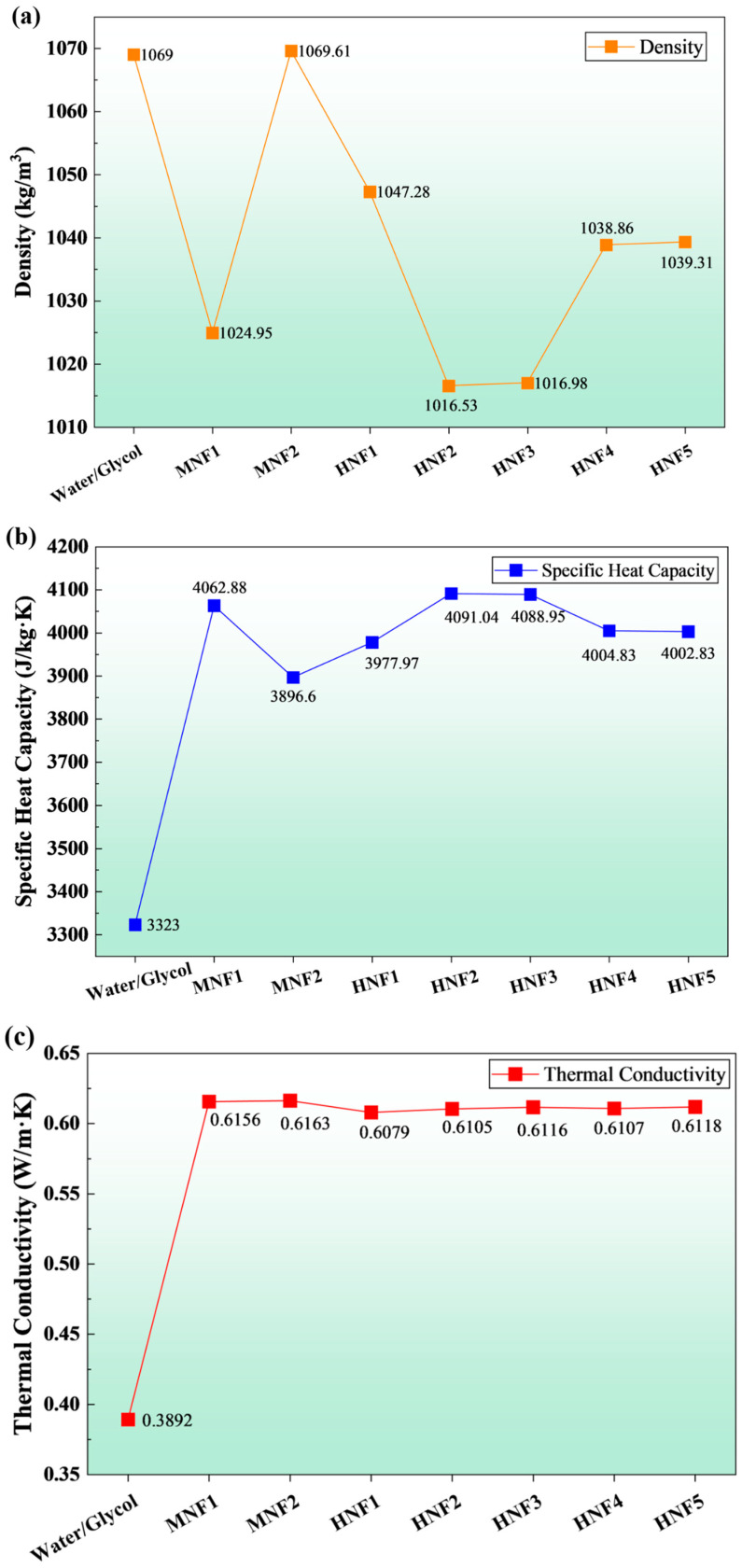

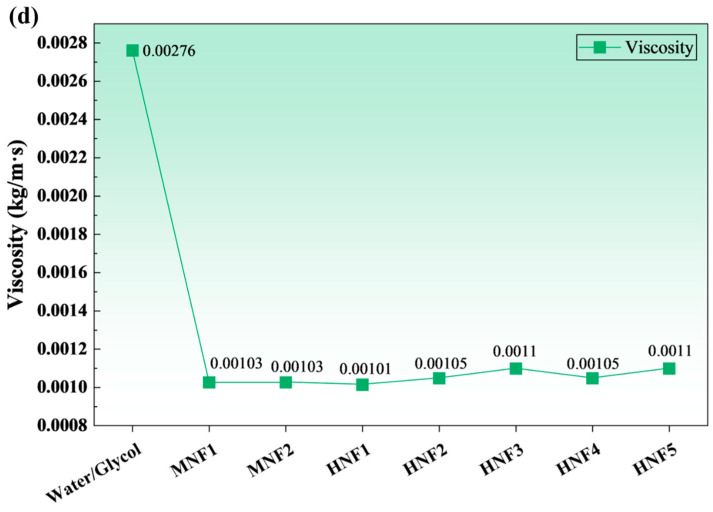
Comparison of thermophysical properties of Water/Glycol and nanofluids: (**a**) Density; (**b**) Specific heat capacity; (**c**) Thermal conductivity, and (**d**) Viscosity.

Figure 6 compares the thermophysical properties of the baseline 50/50 water-glycol (W/G) mixture with the proposed MNFs and HNFs. Relative to W/G, all nanofluids exhibit markedly higher thermal conductivities, ranging from 0.608 to 0.616 W/m·K, compared to 0.389 W/m·K. Improving the thermal conductivity of the nanofluid enhances heat conduction within the coolant and reduces thermal resistance between the microchannel wall and the bulk flow. In addition, the nanofluids exhibit higher specific heat capacities of 3897–4091 J/kg·K, compared with W/G’s 3323 J/kg·K, indicating greater sensible heat storage per unit mass and supporting lower coolant temperature rises for a given heat load.

The density variations among the coolants are comparatively modest. The W/G mixture and Cu-based nanofluids show the highest densities from 1069 to 1070 kg/m^3^, whereas Al_2_O_3_ and carbon-based hybrids yield slightly lower values from 1017 to 1047 kg/m^3^. These density shifts primarily influence the mass flow rate when the inlet condition is specified volumetrically and can therefore affect the heat removal rate of the coolant for the battery module through microchannels. However, the most consequential contrast is observed in viscosity. W/G exhibits a substantially higher dynamic viscosity of 0.00276 kg/m·s than all MNFs and HNFs of 0.00101–0.00110 kg/m·s. This implies a significantly larger hydraulic penalty for W/G at the same volumetric flow rate, consistent with higher pressure drops and pumping requirements in microchannel cooling.

Within the nanofluid set, differences are subtle but physically meaningful. The MNFs provide the largest increase in thermal conductivity, with Cu at 0.6163 W/m·K marginally exceeding Al_2_O_3_ at 0.6156 W/m·K, while the hybrid formulations cluster tightly around 0.608–0.612 W/m·K. Among hybrids, graphene-containing cases exhibit slightly higher viscosity (0.001099 kg/m·s) than MWCNT-containing cases (0.001048 kg/m·s), suggesting greater flow resistance for platelet-like additives at the same loading. Meanwhile, Al_2_O_3_-MWCNT yields the highest heat capacity of 4091 J/kg·K, whereas Cu-based hybrids tend to have slightly lower heat capacities of 4003–4005 J/kg·K. Overall, the property set indicates that the nanofluids offer a combination of enhanced thermal conductivity and heat capacity with viscosities close to water-like values, whereas the 50/50 W/G baseline is characterized by a pronounced viscosity penalty and reduced thermal conductivity, conditions that can suppress convective performance when normalized by pumping power.

### 3.2. Comparison of Heat Transfer Characteristics

#### 3.2.1. Maximum Temperature

**Figure 7:**
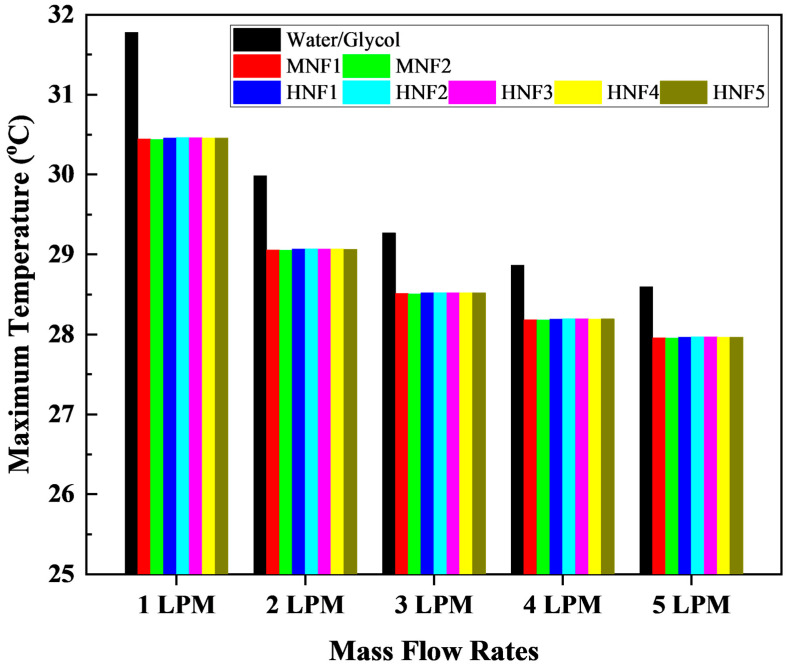
Variation of the maximum temperature of the battery module between Water/Glycol and nanofluids.

Figure 7 presents the variation of the T_max_ of the battery module at a 3C discharge rate for the 50/50 W/G mixture, MNFs, and HNFs over the flow range of from 1 LPM to 5 LPM. For all coolants, T_max_ decreases monotonically with increasing flow rate, reflecting enhanced convective heat removal and reduced thermal resistance at higher Reynolds numbers [11]. For the W/G baseline, T_max_ decreases from 31.78 °C at 1 LPM to 28.60 °C at 5 LPM, corresponding to an overall reduction of approximately 3.2 °C.

At each mass flow rate, both MNFs and HNFs consistently produce lower T_max_ than W/G. At 1 LPM, the T_max_ for nanofluids is approximately 30.44–30.46 °C, representing a reduction of about 4.1% relative to W/G. This improvement can be attributed primarily to the substantially higher thermal conductivity and specific heat capacity of the nanofluids compared with W/G, which enhance heat diffusion near the channel wall and increase the coolant’s sensible heat absorption capacity [27]. As the flow rate increases, the T_max_ gap between W/G and nanofluids gradually narrows, falling to approximately 0.6–0.7 °C at 5 LPM. This diminishing difference is physically consistent with convection-dominated heat transfer at higher flow rates, where the relative contribution of thermal conductivity becomes less pronounced, and the thermal resistance is increasingly governed by bulk convection [58].

The differences of T_max_ among MNFs and HNFs are comparatively small, with lower than 0.05 °C at most operating points. These results indicate that, under the present nanoparticle loadings of 0.9% mono and 0.45%/0.45% hybrid, the enhancement in the macroscopic peak temperature is more influenced by the overall improvement in effective thermophysical properties than by the specific nanoparticle combination. Notably, all cooling configurations maintain T_max_ well within the recommended Lithium-ion battery operating range of 25–40 °C, ensuring safe thermal operation under 3C discharge [3,19]. However, the consistently lower peak temperatures achieved by the nanofluids suggest an improved thermal safety margin and potentially reduced thermal degradation rates compared with the conventional W/G coolant. Generally, the results demonstrate that nanofluid coolants provide measurable reductions in T_max_ of the battery module, particularly at lower flow rates where conductive and thermophysical enhancements play a more dominant role.

**Figure 8:**
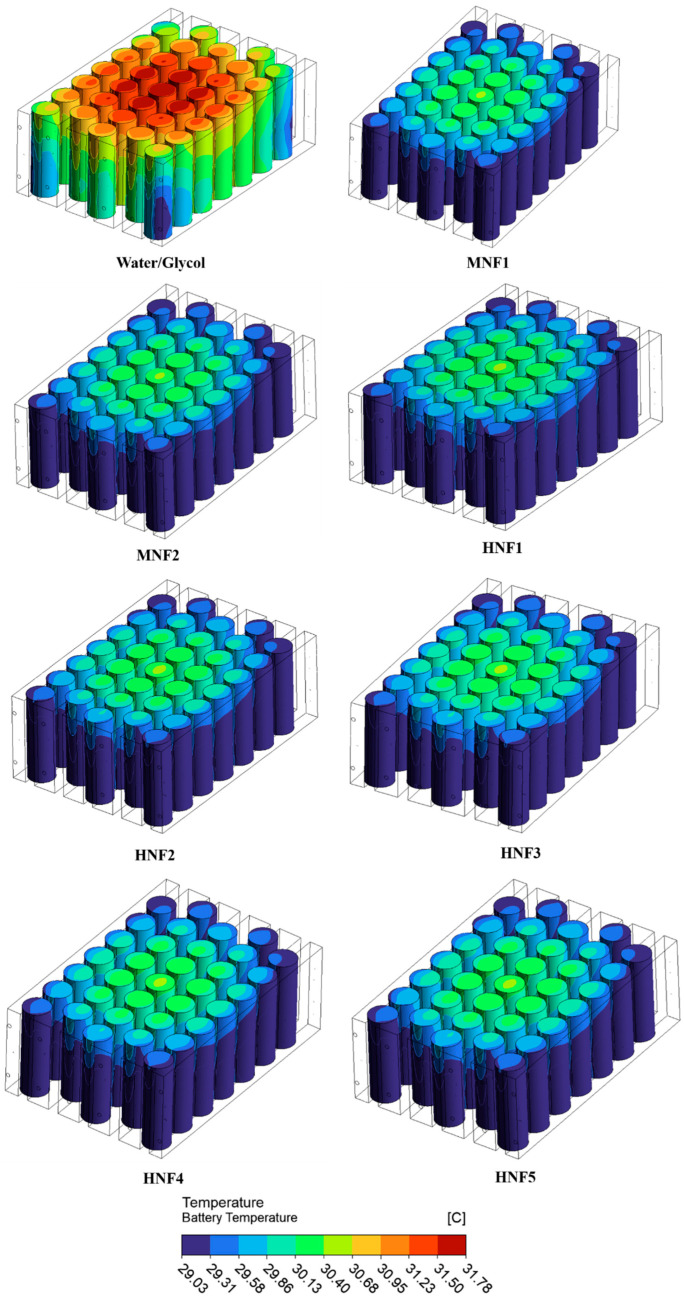
Temperature distribution of the battery module between Water/Glycol and nanofluid cooling.

The temperature contours in Figure 8 illustrate the spatial temperature distribution within the battery module, cooled by the conventional water-glycol (50/50) coolant and various mono- and hybrid-nanofluids at a coolant flow rate of 1 LPM. As observed, the water-glycol coolant results in a relatively higher-temperature region concentrated near the module’s central cells, indicating less effective heat removal from the battery core, where heat generation is typically highest. In contrast, the nanofluid coolants exhibit a more uniform temperature distribution across the module, with noticeably lower peak temperatures and reduced thermal gradients. This improvement can be attributed to the enhanced thermophysical properties of nanofluids, particularly their higher thermal conductivity and improved convective heat transfer capability, which facilitate more efficient heat dissipation from the battery surfaces to the coolant flowing through the microchannels. Consequently, the use of nanofluids leads to better temperature uniformity within the battery pack, which is essential for maintaining consistent electrochemical performance, reducing thermal stress, and improving the operational safety and lifespan of lithium-ion battery systems.

#### 3.2.2. Temperature Difference

**Figure 9:**
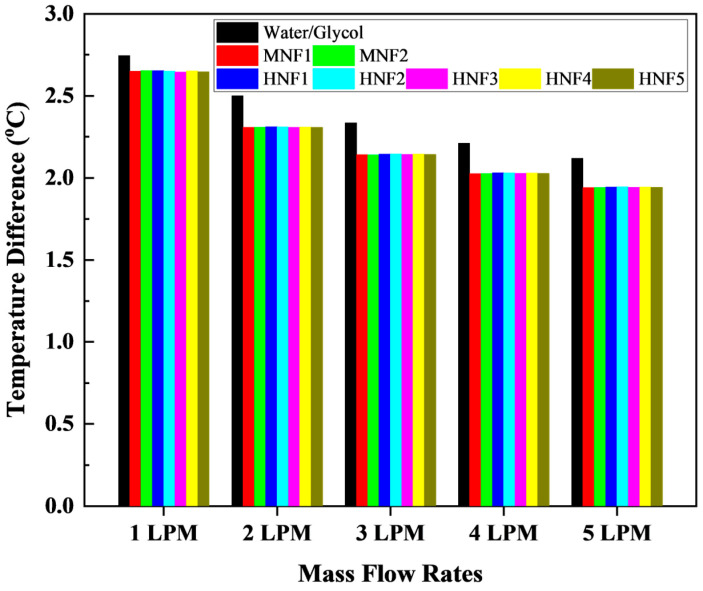
Variation of the temperature difference of the battery module between Water/Glycol and nanofluids.

Figure 9 compares the ΔT of the battery module for the W/G mixture with those of the proposed MNFs and HNFs at a 3C discharge rate. In battery thermal management, maintaining a low ΔT of the module is critical because non-uniform temperatures accelerate cell-to-cell imbalance, amplify localized aging rates, and may promote the onset of safety-critical hotspots [59]. Consequently, lithium-ion battery modules are commonly designed to keep the ΔT below 5 °C to preserve performance, lifespan, and operational safety [60]. Against this criterion, all cases in the present study satisfy the uniformity requirement, with ΔT ranging from approximately 1.94 °C to 2.75 °C across the investigated flow rates.

An apparent monotonic decrease in ΔT with increasing flow rate is observed for all coolants, indicating that more vigorous forced convection improves both peak temperature suppression and temperature homogenization. For W/G, ΔT decreases from 2.745 °C at 1 LPM to 2.119 °C at 5 LPM, representing a 23% reduction, reflecting more effective heat extraction and a reduced axial rise in coolant temperature along the microchannel length at higher flow rates. Importantly, the nanofluids achieve consistently lower ΔT than W/G at every operating point. At 1 LPM, the nanofluids reduce ΔT to 2.645–2.653 °C, corresponding to an absolute improvement of roughly 0.09–0.10 °C compared with W/G. Although this reduction is modest in magnitude, it is systematic. It indicates enhanced thermal spreading and more uniform convective cooling of the cell array, particularly in regions furthest from the coolant inlet, where coolant warming and reduced driving temperature difference typically lead to higher local cell temperatures.

As the flow rate increases, the advantage of nanofluids becomes more pronounced relative to W/G. At 5 LPM, W/G yields ΔT of 2.119 °C, whereas the nanofluids produce ΔT from 1.940 to 1.944 °C, an improvement of about 8.0% to 9.0%. This behavior suggests that even when bulk convection is strong, the higher effective thermal conductivity and heat capacity of nanofluids continue to support more uniform wall-to-bulk heat transport and reduce the spatial gradients driven by localized heat generation within the battery module. In practical terms, a lower ΔT improves cell-to-cell consistency during high C-rate discharge, which helps mitigate imbalance growth and maintain usable capacity during aggressive duty cycles [61].

Differences of ΔT among MNFs and HNFs remain comparatively small, implying that, at the present loadings, temperature uniformity is governed primarily by the global thermo-hydraulic conditions, including flow rate and channel network, and secondarily by fine variations in nanofluid properties. Nevertheless, a slight trend is observed that the lowest ΔT values are achieved by the nanofluids at higher flow rates, with 1.94 °C at 5 LPM, consistent with their generally lower viscosity and enhanced thermal properties. Overall, these results indicate that replacing W/G with either mono- or hybrid-nanofluids can improve thermal uniformity while remaining comfortably below the 5 °C uniformity target widely adopted for lithium-ion modules, thereby supporting improved performance stability, slower degradation, and a higher safety margin under 3C discharge rate operation.

#### 3.2.3. Heat Transfer Coefficient

**Figure 10:**
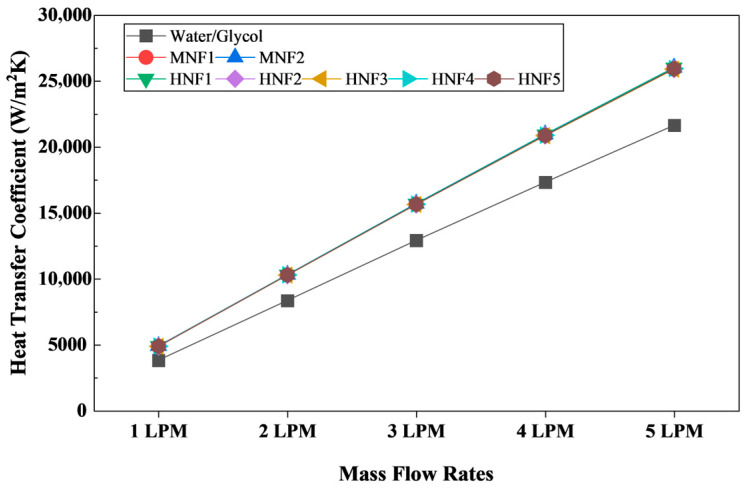
Variation of the heat transfer coefficient between Water/Glycol and nanofluids during cooling for a battery module.

Figure 10 summarizes the average heat transfer coefficient (HTC) obtained for the W/G mixture and for the MNFs and HNFs at a 3C discharge rate over the flow range from 1 to 5 LPM. For all working fluids, HTC increases monotonically with increasing flow rate, rising from 3824 W/m^2^·K at 1 LPM to 21,644 W/m^2^·K at 5 LPM for W/G. This trend is expected because increasing the volumetric flow rate elevates the bulk velocity and Reynolds number, thereby thinning the hydrodynamic and thermal boundary layers and increasing convective heat transfer at the coolant-wall interface [43]. In addition, a higher mass flow rate increases the coolant’s heat removal capacity while limiting the rise in bulk fluid temperature, thereby sustaining a larger driving temperature difference along the channel length.

Compared with W/G, all nanofluids provide a substantial enhancement in HTC across the entire operating envelope. At 1 LPM, the average HTC increases from 3824 W/m^2^·K of W/G to approximately 4863–4900 W/m^2^·K for the nanofluids, corresponding to an improvement of roughly 27–28%. The enhancement remains significant at higher flow rates at 5 LPM, nanofluids yield 25,880–26,208 W/m^2^·K compared to 21,644 W/m^2^·K for W/G, an improvement of approximately 20–21%. The persistence of this advantage indicates that, even when convection becomes dominant at high flow rates, the nanofluids retain a measurable capacity to reduce the effective thermal resistance. Mechanistically, this behavior is consistent with their higher thermal conductivity and heat capacity, as well as their substantially lower viscosity than W/G, which can yield higher Reynolds numbers at the same volumetric flow rate and thus stronger convection.

Among the nanofluids, the mono formulations provide the highest HTC values, with MNF2 marginally outperforming MNF1 at all flow rates. For instance, at 3 LPM, MNF2 achieves 15,709 W/m^2^·K compared with 15,672 W/m^2^·K for MNF1. This small but consistent difference can be attributed to the slightly higher thermal conductivity of the Cu-based nanofluid, which reduces the thermal resistance at the coolant-wall interface. The hybrid nanofluids form a narrow performance band, indicating that at the investigated total loading, the macroscopic convective enhancement is relatively insensitive to the specific hybrid pairing. Nevertheless, minor distinctions are observable that HNF1 tends to yield the highest HTC among hybrids, reaching 15,688 W/m^2^·K at 3 LPM and 26,021 W/m^2^·K at 5 LPM, whereas HNF4 and HNF5 are slightly lower at high flow rates. Such variations are consistent with small differences in effective thermal conductivity and viscosity between graphene- and MWCNT-based formulations. Graphene hybrids typically exhibit slightly higher thermal conductivity but may also introduce a marginal increase in viscosity, and the net effect depends on the balance between improved thermal diffusion and flow resistance-induced changes in local convection.

From an engineering standpoint, the results indicate that replacing W/G with either mono- or hybrid-nanofluids can significantly increase convective heat transfer coefficients during high C-rate discharge, thereby supporting lower module peak temperatures and improved temperature uniformity. Importantly, the relative improvement in HTC is larger at low flow rates, suggesting that nanofluids are particularly beneficial when pump capacity is constrained or when system-level packaging limits the achievable coolant flow. However, since HTC alone does not capture the hydraulic penalty, the discussion of convective enhancement should be interpreted together with pressure drop, friction factor, and overall performance evaluation criteria (PEC) in subsequent sections to establish a complete thermo-hydraulic assessment.

#### 3.2.4. Nusselt Number

**Figure 11:**
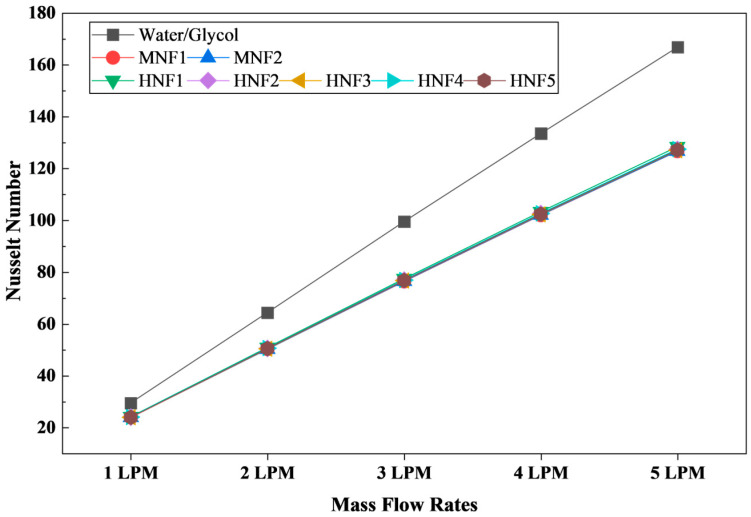
Variation of the Nusselt number between Water/Glycol and nanofluids during cooling for a battery module.

Figure 11 reports the average Nusselt number (Nu) for the W/G mixture and for MNFs and HNFs over the investigated flow range from 1 to 5 LPM under the 3C discharge condition. As expected for internal forced convection, Nu increases monotonically and strongly with increasing flow rate for all coolants, reflecting the progressive strengthening of convective transport as inertial effects grow and the thermal boundary layer becomes thinner [62]. For the W/G case, Nu increases from 29.47 at 1 LPM to 166.83 at 5 LPM, indicating a substantial enhancement of convective heat transfer with flow rate. The same qualitative trend is observed for all nanofluids. However, the magnitude of Nu is consistently lower than that of W/G at each operating point.

The observed reduction in nanofluid Nu relative to W/G is physically meaningful and does not contradict the higher HTCs reported in Section 3.2.3. By definition, Nu = hD/k, where k is the thermal conductivity of the coolant. The increased thermal conductivity of nanofluids directly increases the HTC. However, it also increases the denominator in the Nu formulation. As a result, the normalized heat transfer performance (Nu) may decrease even when the actual HTC improves. In other words, the nanofluids enhance wall heat removal primarily through improved near-wall heat diffusion and increased heat-carrying capacity, rather than by dramatically increasing the intensity of convection in a dimensionless sense. This distinction is important for rigorous interpretation: HTC describes the net heat removal capability in the present geometry, while Nu isolates the relative strength of convective transport compared with conduction within the coolant.

Quantitatively, at 1 LPM, Nu for W/G is 29.47, whereas nanofluids yield approximately 23.81–24.11, representing a reduction of roughly 18–19%. At intermediate flow rates of 3 LPM, W/G achieves 99.55 while nanofluids are clustered in the range of 76.37–77.42, a 22–23% lower. At 5 LPM, the gap persists with 166.83 for W/G compared to 126.65–128.41 for nanofluids, a 23–24% lower. The relatively stable proportional difference across the operating envelope suggests that the comparative Nu reduction is systematic and consistent with the property normalization effect, rather than a numerical artifact confined to a specific flow rate.

Differences of Nu among MNFs and HNFs are relatively small, but several patterns can be highlighted. MNF2 exhibits slightly higher Nu than MNF1 at most flow rates, consistent with its marginally higher thermal conductivity and very similar viscosity, which can yield a modest difference in the balance between convection and diffusion. Among the hybrids, HNF1 tends to provide the highest Nu within the hybrid group at low-to-moderate flow rates, while the remaining hybrids form a narrow band with variations typically within approximately 1 Nu unit. This tight clustering indicates that at the present total volume fraction, the dimensionless convective response is not strongly sensitive to the exact hybrid pairing. Instead, it is dominated by the common effects of increased thermal conductivity and modest differences in viscosity, which influence the Reynolds and Prandtl numbers.

From a design perspective, the combined interpretation of Section 3.2.3 and Section 3.2.4 is critical. The higher HTC values obtained for nanofluids demonstrate improved absolute heat extraction capacity and are consistent with the reduced T_max_ and improved temperature uniformity reported earlier. Meanwhile, the lower Nu values indicate that the enhancement mechanism is largely conductivity-driven rather than a fundamental intensification of convective transport when expressed in a dimensionless form. This distinction helps reconcile seemingly counterintuitive outcomes and strengthens the mechanistic understanding of nanofluid performance in microchannel battery thermal management.

#### 3.2.5. Pressure Drop

**Figure 12:**
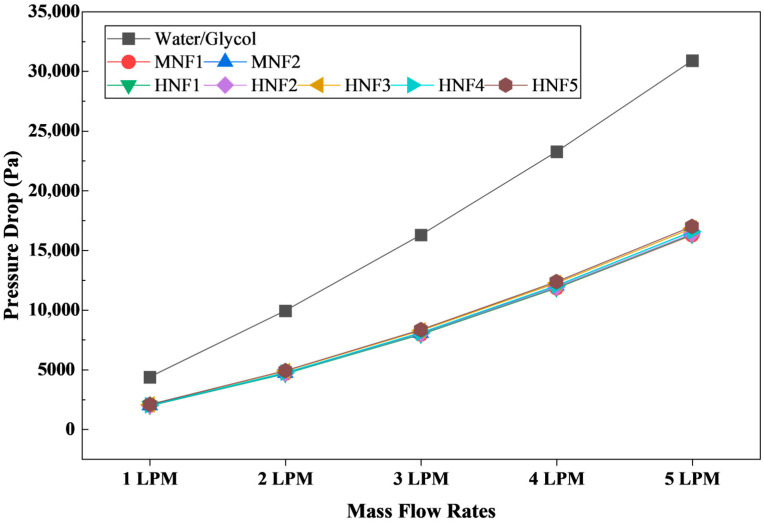
Variation of the pressure drop between Water/Glycol and nanofluids during cooling for a battery module.

Figure 12 compares the pressure drop (Δ*P*) across the microchannel cooling network for the W/G mixture and the MNFs and HNFs at 1–5 LPM. As expected for internal flow in confined passages, Δ*P* increases sharply with increasing flow rate for all coolants because viscous shear and momentum losses intensify as the bulk velocity rises [11,62]. For W/G, Δ*P* grows from 4373.7 Pa at 1 LPM to 30,887 Pa at 5 LPM, indicating that pumping requirements become increasingly significant at high flow rates in the present channel geometry.

A key observation is that all nanofluids produce substantially lower pressure drops than W/G at the same volumetric flow rate. At 1 LPM, Δ*P* decreases from 4373.7 Pa of W/G to approximately 1949.4–2062.5 Pa for the nanofluids, corresponding to a reduction of roughly 53–56%. The same trend persists at higher flow rates at 3 LPM, W/G yields 16,282.8 Pa, whereas nanofluids lie in the range of 7924.7–8326.6 Pa, approximately 49–51% lower. Even at 5 LPM, nanofluid Δ*P* values of 16,254.1–16,991.8 Pa remain close to half of the W/G baseline with 30,887.7 Pa. This pronounced hydraulic advantage is consistent with the much higher viscosity of the W/G mixture of 0.00276 kg/m·s compared with the nanofluids from 0.00101 to 0.00110 kg/m·s. Since pressure drops in microchannels scale strongly with viscosity, the viscosity penalty of W/G directly translates into higher Δ*P*, leading to higher pumping power demand.

Differences among nanofluids are smaller but still interpretable. The mono nanofluids (MNF1 and MNF2) exhibit the lowest Δ*P* overall, with values of 1949.4–1967.3 Pa at 1 LPM and 16,254.1–16,567.2 Pa at 5 LPM. The HNFs exhibit slightly higher Δ*P*, particularly in graphene-containing formulations (HNF3 and HNF5), consistent with their slightly higher viscosity compared with MWCNT-based hybrids. For example, at 5 LPM, HNF5 reaches 16,991.8 Pa, marginally higher than HNF2/HNF4, which ranges from 16,380.3 to 16,556.5 Pa. Although these differences are modest, with lower than 5% within the nanofluid group, they are important when evaluating overall thermo-hydraulic performance because small increases in Δ*P* at high flow rates can translate into noticeable additional pumping power.

From an application standpoint, the Δ*P* results provide a strong rationale for benchmarking nanofluids against W/G rather than pure water in EV battery thermal management. W/G mixtures are widely used in automotive cooling loops for freeze protection and corrosion control, but their elevated viscosity imposes a substantial hydraulic penalty in compact microchannel cold plates. The present results indicate that, at equal volumetric flow rates, the proposed nanofluids can achieve improved thermal performance with lower T_max_, improved temperature uniformity, and higher HTC, while simultaneously reducing Δ*P* by approximately half relative to W/G. This combined benefit directly supports the improved PEC observed when W/G is used as the baseline, since PEC explicitly balances heat transfer augmentation against hydraulic cost, as analyzed in the subsequent section.

#### 3.2.6. Friction Factor

**Figure 13:**
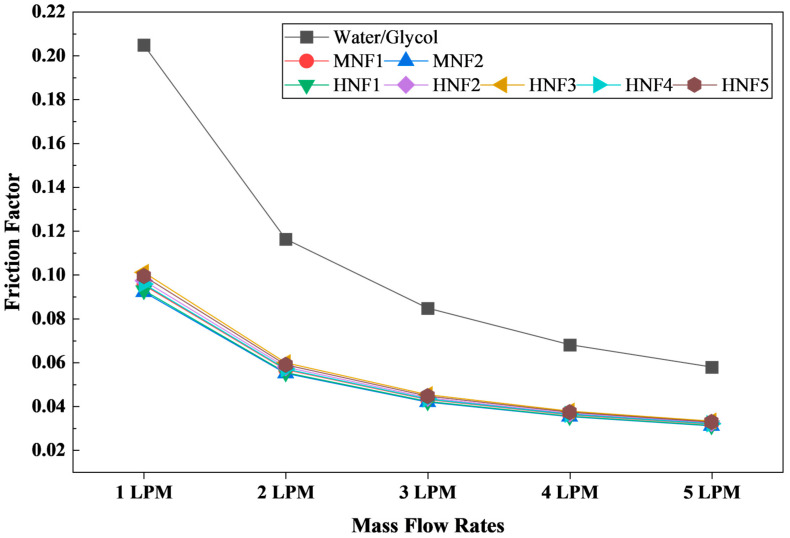
Variation of the friction factor between Water/Glycol and nanofluids during cooling for a battery module.

Figure 13 presents the friction factor (f) for the W/G mixture and for the MNFs and HNFs across the investigated flow range from 1 to 5 LPM. In all cases, f decreases monotonically as the flow rate increases. This behavior is consistent with internal duct flows, where the dimensionless resistance coefficient typically declines with increasing Reynolds number because the pressure drop does not scale as rapidly as the dynamic pressure term embedded in the friction factor definition [58]. Consequently, even though the absolute pressure drop increases with flow rate (Section 3.2.5), the normalized resistance, quantified by f, decreases.

The W/G mixture exhibits the highest f at every operating point, decreasing from 0.205 at 1 LPM to 0.0579 at 5 LPM. In contrast, all nanofluids yield substantially lower values, with MNF1 and MNF2 showing the smallest f overall with approximately 0.095 and 0.092 at 1 LPM, reducing to 0.0318 and 0.0310 at 5 LPM. Therefore, the hydraulic advantage of nanofluids relative to W/G is pronounced, resulting in roughly a 50–55% reduction in the f across the flow range. This finding aligns with the fluid-property comparison, which shows that W/G has a markedly higher viscosity, leading to higher wall shear stress and greater pressure drop for a given velocity field. When expressed in a dimensionless form, the higher viscous contribution in W/G manifests as a higher f.

Within the nanofluid group, the differences of f are comparatively modest but remain physically interpretable. Hybrid nanofluids containing graphene (HNF3 and HNF5) tend to show slightly higher friction factors than the MWCNT-containing hybrids (HNF2 and HNF4) and the MNFs, particularly at lower flow rates. For example, at 1 LPM, HNF3 yields 0.101 compared with 0.095–0.097 for the other hybrids and 0.092–0.095 for the MNFs. This small elevation is consistent with the slightly higher viscosity reported for graphene-based hybrids, which increases shear resistance in confined microchannel flow. Nonetheless, the spread among nanofluids remains relatively small, with values below 10% at most flow rates, indicating that at the present volume fraction, the channel friction is dominated by base-fluid-like behavior rather than by strong rheological divergence between nanoparticle combinations.

From a system perspective, friction factor trends complement the pressure-drop results by providing a Reynolds-number-normalized view of hydraulic losses. The consistently higher f of W/G indicates that it imposes a greater flow resistance penalty in microchannels than either mono- or hybrid-nanofluids at equivalent volumetric flow rates. This result is particularly important for EV thermal management, where pump power consumption affects overall vehicle efficiency.

#### 3.2.7. Performance Evaluation Criteria

**Figure 14:**
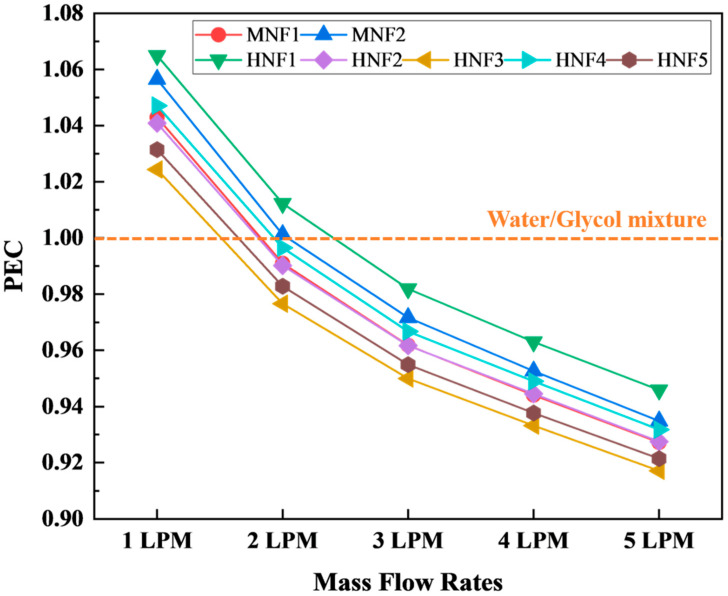
Variation of the PEC between Water/Glycol and nanofluids during cooling for a battery module.

Figure 14 summarizes the PECs for the investigated coolants relative to the W/G mixture as the reference (PEC = 1). PEC provides an integrated thermo-hydraulic metric by balancing heat transfer enhancement with hydraulic penalty, thereby offering a more application-relevant assessment than heat transfer indicators alone. In the present study, PEC values for the nanofluids span approximately 0.917–1.065 across 1–5 LPM, indicating that the relative advantage of nanofluids is strongly dependent on operating flow rate and, consequently, on the interplay between convection strengthening and pressure drop escalation.

It is observed that the maximum PEC occurs at 1 LPM rather than at higher flow rates. At a low flow rate of 1 LPM, all nanofluids achieve PECs above 1, specifically 1.024–1.065, demonstrating a net performance benefit relative to W/G under pump-limited conditions. This behavior is attributed to the competing effects of heat transfer enhancement and pressure drop. Although increasing the flow rate improves convective heat transfer, the associated increase in pressure drop becomes dominant at higher flow rates, resulting in a significant increase in the hydraulic penalty. At 1 LPM, the improved thermal conductivity and heat capacity of nanofluids provide a larger relative benefit, while their lower pressure drop and friction factor than W/G help preserve overall thermo-hydraulic performance. Consequently, 1 LPM provides the most favorable balance and yields the highest PEC. This outcome is consistent with the combined results in Section 3.2.3, Section 3.2.4, Section 3.2.5 and Section 3.2.6, which show that nanofluids increase the heat transfer coefficient while simultaneously reducing pressure drop and friction factor relative to the viscous W/G baseline. The best-performing formulations at 1 LPM are MNF2 and HNF1, with PECs of 1.056 and 1.065, respectively, suggesting that, under weak convection, conductivity-driven heat transfer improvements can be realized without incurring a commensurate increase in hydraulic cost. Practically, this implies that at low volumetric flow rates, representative of energy-efficient pumping strategies, nanofluid coolants can offer an attractive alternative to conventional W/G in microchannel cold plates.

As the flow rate increases to 2 LPM, PEC values approach unity and begin to diverge between the coolants. Several formulations remain marginally above unity, such as PECs of MNF1/MNF2/HNF1 ranging from 1.001 to 1.012, while others fall slightly below, such as PECs of HNF2/HNF5 ranging from 0.98 to 0.99. This near-unity behavior indicates a transitional regime in which the incremental gains in heat transfer are increasingly offset by growing hydraulic demands. Physically, higher velocities intensify inertial losses and can reduce the relative importance of improved thermal conductivity in determining overall convective performance, particularly when the baseline W/G already shows a strong increase in Nu with flow rate.

At mass flow rates of 3–5 LPM, PEC values for all nanofluids drop below unity, reaching 0.96–0.99 at 3 LPM and decreasing further to 0.92–0.95 at 5 LPM. This shift indicates that, at higher flow rates, the thermo-hydraulic advantage of nanofluids relative to W/G diminishes and ultimately reverses when assessed through PEC. A plausible explanation is that the W/G reference experiences a steep rise in Nusselt number with increasing flow rate (Section 3.2.4), while the relative heat-transfer benefit from nanofluids becomes less pronounced under convection-dominated conditions. Simultaneously, although nanofluids still exhibit lower friction factors than W/G, the PEC normalization penalizes hydraulic losses with a sublinear exponent. Thus, the remaining pressure drop difference is insufficient to compensate for the relative reduction in dimensionless convective performance at high flow rates. From a practical standpoint, this suggests that nanofluids are most advantageous when the cooling system is operated at moderate-to-low flow rates, whereas at higher flow rates the performance gap between coolants narrows and W/G may become competitive in a PEC sense. This does not mean that nanofluids lose their cooling capability at high flow rates. Rather, their relative advantage over W/G decreases because the baseline W/G coolant also experiences strong convection enhancement with increasing flow rate.

Comparing formulations, MNF2 and MNF1 are generally among the strongest performers across the range, with MNF2 yielding the highest PEC at 1 LPM and maintaining relatively high values at elevated flow rates, with 0.935 at 5 LPM. Among hybrids, HNF1 shows competitive PEC at a low flow of 1.065 but declines similarly at higher flow. HNF3 exhibits the lowest PEC at high flow with 0.917 at 5 LPM, consistent with its slightly higher viscosity and the associated increase in frictional resistance. Overall, the PEC analysis reinforces an important design implication: when benchmarked against automotive-relevant W/G coolant, nanofluids can provide net thermo-hydraulic benefits in microchannel battery cooling, particularly at low flow rates, while their advantage becomes marginal or even negative at higher flow rates. This operating-point dependence should be emphasized in system-level discussions, as it directly informs pump sizing, control strategies, and coolant selection for EV thermal management.

From a practical implementation perspective, it is important to recognize that the current analysis assumes stable, well-dispersed nanofluids under single-phase conditions. In real microchannel systems, nanoparticle stability remains a critical challenge. Sedimentation and agglomeration of nanoparticles can lead to deposition on channel walls, resulting in particulate fouling, reduced heat transfer efficiency, and potential clogging of microchannels. These effects are particularly significant in compact cooling geometries, where small hydraulic diameters increase the sensitivity to particle accumulation. In addition, long-term interactions between nanoparticles and channel materials may alter surface characteristics, potentially leading to erosion or altered thermal resistance. Previous studies have reported that nanofluid fouling and instability can degrade system performance over time and increase maintenance requirements [53,54]. Therefore, ensuring long-term dispersion stability through appropriate nanoparticle selection, concentration control, and surface treatment is essential for practical applications.

### 3.3. Hybrid Nanofluid with Different Composition Ratios

Building on the PEC screening in Section 3.2.7, HNF1 of Al_2_O_3_-Cu with total volume fraction 0.90% (50/50) operated at 1 LPM was identified as the most favorable nanofluid and flow combination. To further refine this optimum, a targeted parametric study was conducted by varying the Al_2_O_3_/Cu volume fraction ratio while keeping the total nanoparticle concentration constant. Three compositions were examined: 50/50 (0.45–0.45%), 30/70 (0.27–0.63%), and 70/30 (0.63–0.27%). This approach isolates the effect of hybrid composition from that of total loading, thereby enabling a more defensible interpretation of the role of the Cu fraction in thermo-hydraulic performance.

The comparison of the heat transfer characteristics of HNF1 with different composition ratios is presented in Table 5. The results indicate that altering the Al_2_O_3_/Cu ratio produces only marginal changes in the module thermal metrics at 1 LPM. The T_max_ remains essentially invariant with T_max_ of 30.454–30.457 °C, and the module temperature non-uniformity is similarly stable with ΔT of 2.652–2.653 °C. These nearly identical values suggest that, at this total loading and operating condition, the pack-level temperature field is governed primarily by the imposed flow rate and cold plate geometry, while the incremental changes in effective thermophysical properties introduced by shifting the Al_2_O_3_/Cu ratio are second-order. Nevertheless, subtle differences appear in the convective indicator. Specifically, the 30/70 case yields the highest HTC of 4890.933 W/m^2^·K, slightly exceeding the 50/50 case of 4886.012 W/m^2^·K and the 70/30 case of 4886.690 W/m^2^·K. In contrast, the Nusselt number shows a weak decreasing trend as the Cu fraction increases from 24.112 to 24.079. This behavior is consistent with the definition Nu=hD/k. Specifically, if Cu enrichment increases the effective thermal conductivity k more than it increases HTC, the normalized Nu can decrease slightly even though the absolute convective heat transfer capacity improves.

**Figure 15:**
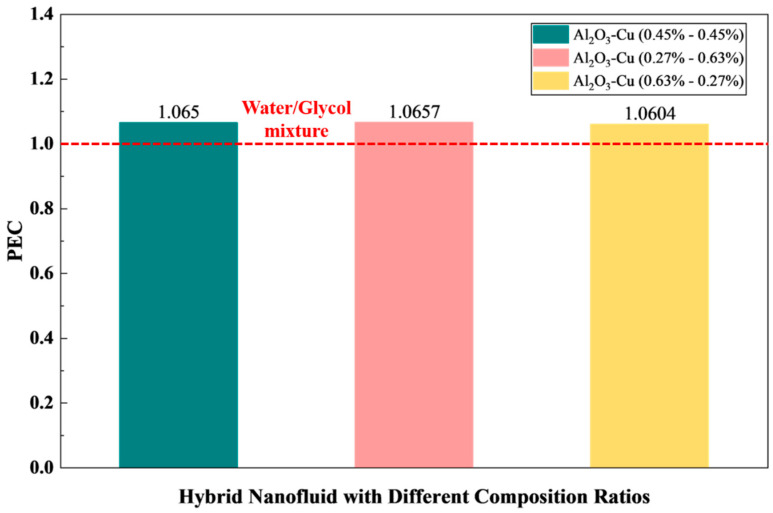
Variation of the PEC for hybrid nanofluid with different composition ratios.

From a hydraulic standpoint, the Δ*P* varies by less than 0.4% across the three compositions, ranging from 1940 to 1947 Pa, and the friction factor remains tightly grouped at 0.0922–0.0935. These small variations indicate that redistributing the hybrid fraction between Al_2_O_3_ and Cu does not materially change the flow resistance at the investigated concentration, which is expected because the effective viscosity changes induced by such small compositional shifts are limited. Importantly, because the PEC formulation combines a heat transfer benefit term with a hydraulics penalty term, the observed PEC differences are mainly driven by small but systematic differences in HTC, Nu, and f. Variation of the PEC for the hybrid nanofluid with different composition ratios is illustrated in Figure 15. The 30/70 mixture achieves the highest PEC of 1.0657, followed closely by the 50/50 mixture of 1.0650, while the 70/30 mixture gives a slightly lower PEC of 1.0604. Although the ranking is consistent, the absolute spread is modest, with a maximum difference of 0.0053 (approximately 0.5%), and therefore the interpretation should be framed as a marginal optimization rather than a step-change improvement. Considering the inherent numerical uncertainty of CFD simulations, the small PEC spread among the tested compositions does not support a strong claim of universal optimality, but it does indicate that the 30/70 case is slightly favorable within the investigated range. In practical terms, the results suggest that a Cu-rich hybrid ratio can offer a small thermo-hydraulic advantage at low flow rate by slightly enhancing HTC without introducing a commensurate increase in pressure drop.

Overall, this composition sweep strengthens the study’s design implications by demonstrating that HNF1 remains robustly favorable at 1 LPM across different Al_2_O_3_/Cu ratios, while identifying Al_2_O_3_-Cu (0.27–0.63%) as the slightly superior formulation under the present conditions. Accordingly, among the tested composition ratios, the 30/70 mixture showed the highest PEC under the present low-flow condition. However, because the maximum PEC difference is only about 0.5%, this result should be interpreted as a marginal performance preference rather than a definitive optimum.

## 4. Conclusions

This study numerically investigated the thermo-hydraulic performance of mono- and hybrid-nanofluids for microchannel cooling of a 21700 cylindrical lithium-ion battery module operating under a 3C discharge condition. A three-dimensional computational model of a 5S7P battery module integrated with microchannel cooling plates was developed, and battery heat generation was predicted using the NTGK electrochemical model. The performance of conventional water-glycol (50/50) coolant was compared with several mono nanofluids of Al_2_O_3_ and Cu and hybrid nanofluids of Al_2_O_3_-Cu, Al_2_O_3_-MWCNT, Al_2_O_3_-Graphene, Cu-MWCNT, and Cu-Graphene over a range of coolant flow rates from 1 to 5 LPM. The following summarizes the main conclusions of the current study:

(a) The results demonstrate that nanofluids significantly improve the thermal performance of the battery cooling system compared with the conventional coolant. All investigated nanofluids effectively reduced the maximum battery temperature and temperature difference within the module while maintaining temperatures within the recommended operating range of 25–40 °C. In addition, nanofluids produced higher heat transfer coefficients and lower pressure drops and friction factors than the water-glycol coolant, due to their enhanced thermophysical properties and lower viscosity.
(b) The results indicate that an optimal flow rate of 1 LPM provides the best thermo-hydraulic performance due to a favorable balance between heat transfer enhancement and pumping power requirements. Specifically, thermo-hydraulic evaluation using PEC revealed that the Al_2_O_3_-Cu hybrid nanofluid (0.45–0.45%) at 1 LPM achieved the best overall cooling performance, with a PEC value of 1.065. Further analysis of hybrid nanoparticle composition ratios showed that a Cu-dominant hybrid mixture of Al_2_O_3_-Cu (0.27–0.63%) provided the highest PEC among the tested composition ratios (1.0657), although the improvement relative to the other tested cases was marginal.
(c) Overall, the findings suggest that hybrid nanofluids represent a promising alternative to conventional water-glycol coolants for microchannel-based BTMSs in EVs, particularly under moderate coolant flow conditions. However, potential practical challenges such as nanoparticle stability, long-term reliability, and economic feasibility should be addressed before large-scale implementation. Future work will focus on experimental validation of the full battery module with nanofluid-based microchannel cooling, as well as a detailed investigation of nanoparticle stability, fouling behavior, and material compatibility to assess long-term system reliability. In addition, future studies should incorporate transient simulations to capture the dynamic thermal response of the battery during charge/discharge cycles.

## Figures and Tables

**Table 1:** Specifications for the 21700-type battery in the present study [12].

Parameters	Value	Unit
Nominal capacity	4.80	Ah
Nominal voltage	3.64	V
Maximum charge voltage	4.20	V
Discharge cut-off voltage	2.50	V
Diameter	21.15 ± 0.2	mm
Height	70.65 ± 0.15	mm
Weight	68.0 ± 1.5	g

**Table 2:** Summary of NTGK correction parameters applied in the simulations.

Coefficient	Value	Coefficient	Value
a_0_	4.194328	b_0_	42.77042
a_1_	−1.23694	b_1_	−414.266
a_2_	3.241781	b_2_	2194.362
a_3_	−12.6903	b_3_	−5228.67
a_4_	19.82467	b_4_	5741.754
a_5_	−10.6283	b_5_	−2358.17
C_1_	1800	C_2_	−0.00095

**Table 3:** Thermophysical properties of water-glycol and nanoparticles [54].

Specifications	Water/Glycol (50/50)	Al_2_O_3_ (Spherical)	Cu (Spherical)	Graphene (Platelets)	MWCNT (Cylindrical)
Density (kg/m^3^)	1069	3970	8933	2200	2100
Specific heat capacity (J/kg·K)	3323	765	385	790	857
Thermal conductivity (W/m·K)	0.3892	40	400	5000	3000
Dynamic viscosity (kg/m·s)	0.00276	-	-	-	-

**Table 4:** Determined the thermophysical properties of both mono and hybrid nanofluids.

Nanoparticle Shape	MNF1	MNF2	HNF1	HNF2	HNF3	HNF4	HNF5
Density (kg/m^3^)	1024.95	1069.61	1047.28	1016.53	1016.98	1038.86	1039.31
Specific heat capacity (J/kg·K)	4062.88	3896.60	3977.97	4091.04	4088.95	4004.83	4002.83
Thermal conductivity (W/m·K)	0.6156	0.6163	0.6079	0.6105	0.6116	0.6107	0.6118
Dynamic viscosity (kg/m·s)	0.001026	0.001026	0.001014	0.001048	0.001099	0.001048	0.001099

**Table 5:** Compare the heat transfer characteristics of HNF1 with different composition ratios.

Properties	50/50 Al_2_O_3_-Cu (0.45–0.45%)	30/70 Al_2_O_3_-Cu (0.27–0.63%)	70/30 Al_2_O_3_-Cu (0.63–0.27%)
Maximum Temperature (°C)	30.457	30.454	30.456
Temperature Difference (°C)	2.653	2.653	2.652
Heat Transfer Coefficient (W/m^2^·K)	4886.012	4890.933	4886.69
Nusselt Number	24.112	24.079	24.068
Pressure Drop (Pa)	1942.42	1947.05	1940.08
Friction Factor	0.0928	0.0922	0.0935
PEC	1.0650	1.0657	1.0604

## Data Availability

The data presented in this study will be available on request to the corresponding author.

## References

[1] Hwang S.G., Lee M.Y., Ko B.S. (2025). Numerical Analysis on Cooling Performances for Connectors Using Immersion Cooling in Ultra-Fast Chargers for Electric Vehicles. Symmetry.

[2] Jiang Z., Ma R., Hou Z., Xuan W., Xu C., Yu B., Wang D., Shi J., Chen J. (2026). Experimental investigation of a vehicle-scale immersion cooling battery thermal management system for high-energy-density battery packs under cooling and heating conditions. Energy.

[3] Tai L.D., Garud K.S., Lee M.Y. (2025). Experimental Study on Thermal Management of 5S7P Battery Module with Immersion Cooling Under High Charging/Discharging C-Rates. Batteries.

[4] Jiang Z., Ma R., Xuan W., Xu C., Yu B., Wang D., Gu T., Shi J., Chen J. (2026). Immersion cooling battery thermal management system design and optimization for high-energy-density battery packs: A comparative study with side cooling plates. Int. J. Heat Mass Transf..

[5] Kureshi D.A., Nandan R., Das M.K. (2026). Improved temperature uniformity and fast charging of high energy density Li-ion battery module via two-phase immersion cooling. Appl. Therm. Eng..

[6] Qian L., Xiao W., Fei G., Xia C., Yi Y., Ma T., Chen S. (2026). Immersion cooling control for ununiform degraded lithium-ion batteries under fast charging. J. Energy Storage.

[7] Tai L.D., Lee M.Y. (2025). Advances in the Battery Thermal Management Systems of Electric Vehicles for Thermal Runaway Prevention and Suppression. Batteries.

[8] Li Y., Bai M., Zhou Z., Wu W., Lv J., Gao L., Huang H., Li Y., Song Y. (2023). Experimental studies of reciprocating liquid immersion cooling for 18650 lithium-ion battery under fast charging conditions. J. Energy Storage.

[9] Shan W., He Q., Cao Z., Zhang Z., Dai Z., Zheng L., Li X. (2026). Numerical study of multi-nozzle inlet structure optimization for immersion cooling systems of large-scale lithium-ion battery pack. J. Energy Storage.

[10] Bai H., Li X., Zhou Z., Zhang Y., Jin Z., Wang H., Zheng P., Zhou X. (2026). Efficient thermal management of lithium-ion battery modules using a simplified two-phase immersion cooling system. Appl. Therm. Eng..

[11] Le T.D., Lee M. (2026). Optimization of thermal performance characteristics for 21,700 lithium-ion battery pack utilizing single-phase immersion cooling: A multi-parameter numerical investigation. Int. Commun. Heat Mass Transf..

[12] Le T.D., Bang Y.M., Nguyen N.H., Lee M.Y. (2025). Artificial Neural Network-Based Optimization of an Inlet Perforated Distributor Plate for Uniform Coolant Entry in 10 kWh 24S24P Cylindrical Battery Module. Symmetry.

[13] Vikram S., Sadar A., Rakshit D. (2026). Experimental study of dielectric coolant-based immersion cooling technique for lithium-ion battery packs. J. Energy Storage.

[14] Lu Y., Sun B., Li L., Huang F., Chen C. (2025). Numerical investigation of vertical vibration effects on immersion cooling heat transfer for lithium-ion battery at high discharge rate. Case Stud. Therm. Eng..

[15] Hyun S.W., Jeong H.J., Kim J.H., Shin D.H. (2026). Two-phase immersion cooling with microcapsule phase-change material particle slurries for heat-transfer enhancement in battery thermal management. Appl. Therm. Eng..

[16] Liu J., Tao L., Yang Q., Wang J. (2025). Recent advances in immersion cooling for thermal management of lithium-ion batteries. Renew. Sustain. Energy Rev..

[17] Miao Y., Gao Y., Liu X., Liang Y., Liu L. (2024). Analysis of State-of-Charge Estimation Methods for Li-Ion Batteries Considering Wide Temperature Range. Energies.

[18] Kouhestani H.S., Liu L., Wang R., Chandra A. (2023). Data-driven prognosis of failure detection and prediction of lithium-ion batteries. J. Energy Storage.

[19] Tai L.D., Garud K.S., Hwang S.G., Lee M.Y. (2024). A Review on Advanced Battery Thermal Management Systems for Fast Charging in Electric Vehicles. Batteries.

[20] Sheng L., Wang H., Zhang C., Zhang X. (2026). Experimental study on immersion cooling performance of a lithium-ion battery module for commercial/industrial energy storage at different ambient temperatures. Int. J. Therm. Sci..

[21] Chen H., Yang Q., Hou Z., Liu J. (2025). Cooling effectiveness of the immersion cooling on the overcharging lithium-ion batteries. Therm. Sci. Eng. Prog..

[22] Liang C., Lai X., Shen K., Zheng Y., Xu C., Peng Y., Li J., Wang X., Wang Y., Hong Y. (2026). Experimental study on immersion cooling for delaying thermal runaway propagation in lithium-ion battery modules. Process Saf. Environ. Prot..

[23] Choi H., Jun Y., Chun I., Lee Y., Lee H. (2026). Data-driven machine learning framework for thermal performance prediction and control of mini-channel and metal-foam assisted battery immersion cooling systems. Energy.

[24] Ding X., Wang Y., Yuan X., Khan D.A., Gu J., Yang Z. (2026). Design of cold plate structures for energy storage battery cooling and analysis of heat transfer performance. Therm. Sci. Eng. Prog..

[25] Kong L., Li Y., Kong L., Chang L., Kou G., Sun J., Mu M. (2026). A novel thermal management system combining phase change material, terminal-cooling with novel arc-fins for a lithium-ion battery pack and the parametric study of the cold plate. J. Energy Storage.

[26] Abdulah A., Kristiawan B., Juwana W.E. (2026). Improving cooling efficiency of rectangular li-ion batteries using hybrid-nanofluid-based mini-channel cold plates. Results Eng..

[27] Jha P., Hussain M., Khan M.K. (2024). Numerical evaluation of nanofluid-based indirect liquid cooling of a Li-ion battery pack using equivalent circuit model under static and dynamic loading conditions. Int. Commun. Heat Mass Transf..

[28] Venkateswarlu B., Chavan S., Woo Joo S., Chul Kim S. (2023). A numerical study on heat transfer performance using nanofluids in liquid cooling for cylindrical battery modules. J. Mol. Liq..

[29] Tousi M., Sarchami A., Kiani M., Najafi M., Houshfar E. (2021). Numerical study of novel liquid-cooled thermal management system for cylindrical Li-ion battery packs under high discharge rate based on AgO nanofluid and copper sheath. J. Energy Storage.

[30] Wankhede S., Pingale A., Patil S., Shahane K. (2025). Experimental study of a novel nanofluid cooling system based on fly ash and serpentine channels for modular lithium-ion battery thermal control. Next Energy.

[31] Yang Z., Wang Y., Yuan X., Ding X., Yang G., Yang X., Li Q. (2026). Staggered counterflow rectangular microchannel liquid-cooled plate based on nanofluids for enhanced heat transfer performance. Int. J. Therm. Sci..

[32] Selvarajoo K., Wanatasanappan V.V., Luon N.Y., Kumar A., Paramasivam P., Agrawal A., Ayanie A.G. (2025). Cooling performance of 18650 lithium-ion battery module using Al_2_O_3_-GO hybrid and mono nanofluids: A numerical study with experimental validation. Case Stud. Therm. Eng..

[33] Wang J., Liu B., Ding X., Li Q., Yu B., Desideri U. (2025). Multi-objective topology optimization design of nanofluid cooling plate for thermal management of lithium ion battery pack. Energy.

[34] Deng J., Hu Z., Chen J., Deng T., Zhang Y., Bai Z., Huang L., He F. (2025). The enhanced cooling effect and critical control capability of nanofluids on suppressing thermal runaway of lithium-ion batteries. J. Energy Storage.

[35] Jongpluempiti J., Vengsungnle P., Poojeera S., Eiamsa-ard S., Naphon N., Srichat A., Manatura K., Naphon P. (2025). Thermal profile analysis of 18650 Li-ion battery module with embedded copper foam in the nanofluid cooling jacket. Case Stud. Chem. Environ. Eng..

[36] Kanti P.K., Yang E.S.J., Wanatasanappan V.V., Sharma P., Said N.M. (2024). Impact of hybrid and mono nanofluids on the cooling performance of lithium-ion batteries: Experimental and machine learning insights. J. Energy Storage.

[37] Hasan H.A., Togun H., Abed A.M., Mohammed H.I., Armaghani T. (2025). Cooling lithium-ion batteries with silicon dioxide -water nanofluid: CFD analysis. Renew. Sustain. Energy Rev..

[38] Venkateswarlu B., Chavan S., Joo S.W., Kim S.C., Nisar K.S. (2025). A numerical investigation of heat transfer performance in a prismatic battery cooling system using hybrid nanofluids. Case Stud. Therm. Eng..

[39] Esmaeili Z., Sheikholeslami M. (2025). Enhanced thermal management of lithium-ion batteries using hybrid nanofluids in finned mini-channels: Energy and entropy analyses. Eng. Sci. Technol. Int. J..

[40] Banerjee R., Nidhul K. (2025). Immersion cooling performance of nanofluid in a cylindrical cell battery pack using two-phase numerical simulations for varying discharge rates: A comparative study. Results Eng..

[41] Maurya N., Bhattacharyya S., Khatri S., Goel V. (2025). Impact of magnetic fields on magnetic nanofluid heat transfer in enhanced mini-channels for high-performance cooling. Int. J. Heat Mass Transf..

[42] Babar H., Wu H., Eltaweel M., Zhang W. (2025). Performance evaluation of nanofluid-enhanced biomimetic liquid-cooled heat sinks for efficient thermal management applications. Int. J. Heat Mass Transf..

[43] Yogeshwar D., Repaka R., Marath N.K. (2025). A double serpentine channel liquid cooling plate for hotspot targeted cooling of lithium-ion batteries in a battery module. Int. J. Therm. Sci..

[44] Sarchami A., Tousi M., Kiani M., Arshadi A., Najafi M., Darab M., Houshfar E. (2022). A novel nanofluid cooling system for modular lithium-ion battery thermal management based on wavy/stair channels. Int. J. Therm. Sci..

[45] Jahanbakhshi A., Nadooshan A.A., Bayareh M. (2022). Cooling of a lithium-ion battery using microchannel heatsink with wavy microtubes in the presence of nanofluid. J. Energy Storage.

[46] Bao R., Wang Z., Gao Q., Yang H., Zhang B., Tuo Z., Chen S. (2025). Enhancing the thermal management of 21,700 batteries via synergistic porous phase change material and immersion liquid cooling. Int. Commun. Heat Mass Transf..

[47] You N., Chinnasamy V., Lee M., Cho H. (2025). Correlation analysis between design parameters and cooling performance in 21700 battery module using immersion cooling. Therm. Sci. Eng. Prog..

[48] Heidarshenas B., Aghaei A., Hossein Zamani A., Yuan Y. (2025). Comparison of different cooling techniques for a lithium-ion battery at various discharge rates using electrochemical thermal modeling. Appl. Therm. Eng..

[49] Wagh V.A., Saha S.K. (2026). On estimating critical channel number of hybrid battery thermal management system combining phase change material and forced convective immersion cooling. Int. Commun. Heat Mass Transf..

[50] Chen Q., Chen X., Li Z. (2023). A fast numerical method with non-iterative source term for pseudo-two-dimension lithium-ion battery model. J. Power Sources.

[51] Khalili H., Khalilian M., Chitsaz A. (2026). Battery degradation assessment and convective heat transfer estimation in battery cooling systems using surrogate artificial neural networks. Results Eng..

[52] Suresh Patil M., Seo J., Lee M. (2021). A novel dielectric fluid immersion cooling technology for Li-ion battery thermal management. Energy Convers. Manag..

[53] Kumar K., Sarkar J., Mondal S.S. (2025). Energy, exergy, and economic evaluations of various cylindrical lithium-ion battery thermal management systems. Int. Commun. Heat Mass Transf..

[54] Kumar K., Sarkar J., Mondal S.S. (2024). Analysis of ternary hybrid nanofluid in microchannel-cooled cylindrical Li-ion battery pack using multi-scale multi-domain framework. Appl. Energy.

[55] Xin S., Wang C., Xi H. (2023). Thermal management scheme and optimization of cylindrical lithium-ion battery pack based on air cooling and liquid cooling. Appl. Therm. Eng..

[56] Li Y., Zhou Z., Hu L., Bai M., Gao L., Li Y., Liu X., Li Y., Song Y. (2022). Experimental studies of liquid immersion cooling for 18650 lithium-ion battery under different discharging conditions. Case Stud. Therm. Eng..

[57] Kong D., Peng R., Ping P., Du J., Chen G., Wen J. (2020). A novel battery thermal management system coupling with PCM and optimized controllable liquid cooling for different ambient temperatures. Energy Convers. Manag..

[58] Park J.S., Tai L.D., Lee M.Y. (2025). Numerical Study on the Heat Transfer Characteristics of a Hybrid Direct–Indirect Oil Cooling System for Electric Motors. Symmetry.

[59] Koucheh A.B., Sharbati P., Ünlü C., Koşar A., Sadaghiani A. (2026). Comprehensive evaluation of battery cooling mechanisms including two-phase immersion with 3 M™ NOVEC™-7000 and 7100. Energy Convers. Manag..

[60] Yaqteen M.A., Moon S., Kim J.S. (2025). A novel spray-based immersion cooling for Li-ion batteries: An experimental comparison with flow immersion. Appl. Therm. Eng..

[61] Wang D., Kong D., Ping P., Zhao X., Dai X., Ren J., Ren X., Guo J. (2025). Synthetic ester immersion cooling for lithium-ion batteries: A comparison of electro-thermal balancing under static and dynamic conditions and heat transfer analysis. J. Energy Storage.

[62] Dehbozorgi F., Jafarpur K., Rad E.G. (2024). Natural Convection Immersion Cooling of the Cylinders in Nanofluids: Developing a New Nusselt Number Correlation. Iran. J. Sci. Technol. Trans. Mech. Eng..

